# Morphological and phylogenetic analyses reveal two new species of *Coniochaeta* (*Coniochaetaceae*, *Coniochaetales*) from northern Thailand

**DOI:** 10.3897/mycokeys.138.197253

**Published:** 2026-08-05

**Authors:** Chanokned Senwanna, Saowaluck Singsalood, Pratthana Kodchasee, Saowaluck Tibpromma, Jaturong Kumla, Nakarin Suwannarach

**Affiliations:** 1 Office of Research Administration, Chiang Mai University, Chiang Mai 50200, Thailand Center for Yunnan Plateau Biological Resources Protection and Utilization & Yunnan International Joint Laboratory of Fungal Sustainable Utilization in South and Southeast Asia, College of Biology and Food Engineering, Qujing Normal University Qujing China; 2 Center of Excellence in Microbial Diversity and Sustainable Utilization, Chiang Mai University, Chiang Mai 50200, Thailand Center of Excellence in Microbial Diversity and Sustainable Utilization, Chiang Mai University Chiang Mai Thailand; 3 Department of Biology, Faculty of Science, Chiang Mai University, Chiang Mai 50200, Thailand Department of Biology, Faculty of Science, Chiang Mai University Chiang Mai Thailand; 4 Center for Yunnan Plateau Biological Resources Protection and Utilization & Yunnan International Joint Laboratory of Fungal Sustainable Utilization in South and Southeast Asia, College of Biology and Food Engineering, Qujing Normal University, Qujing, China Office of Research Administration, Chiang Mai University Chiang Mai Thailand

**Keywords:** New taxa, phylogeny, pleomorphic fungi, taxonomy, tropical area

## Abstract

*Coniochaeta* is a cosmopolitan fungal genus found in diverse habitats worldwide. During an investigation of microfungi from rock and soil samples collected in northern Thailand between 2024 and 2025, six strains of *Coniochaeta* were isolated. These strains were identified based on morphological characteristics and phylogenetic analyses using sequences of the nuclear ribosomal large subunit (LSU), internal transcribed spacer (ITS), and translation elongation factor 1-alpha (*tef1-α*) regions. These six strains, representing two new species, *Coniochaeta saxicola* and *C. terricola*, are described herein. Full descriptions, illustrations, and a phylogenetic tree showing their phylogenetic positions are provided. In addition, the new taxa are compared with closely related taxa.

## Introduction

*Coniochaeta* is a pleomorphic fungal genus belonging to the family *Coniochaetaceae* (*Coniochaetales*, *Sordariomycetes*, *Ascomycota*) (García et al. 2006; Kabtani et al. 2022). The genus was typified by *Coniochaeta ligniaria*, which is known for its sexual morph (Cooke 1887). Later, Weber et al. (2002) established the link between the *Coniochaeta* sexual morph and the *Lecythophora* asexual morph based on phylogenetic evidence. Following the one fungus–one name principle, the use of *Coniochaeta* over *Lecythophora* has been recommended (Khan et al. 2013; Réblová et al. 2016). The sexual morph of the genus is characterized by dark brown to black, setose ascomata that can be pyriform and ostiolate or globose and non-ostiolate; inamyloid asci; and ellipsoid, dark brown, smooth ascospores with a longitudinal germ slit (García et al. 2006; Asgari et al. 2007). The asexual morph produced phialidic conidiogenous cells (Checa et al. 1988; Damm et al. 2010; Khan et al. 2013). Currently, more than 100 *Coniochaeta* epithets are listed in Index Fungorum (2026) and MycoBank (2026); however, only 92 species have sequence data available (Tan et al. 2025a, 2025b; Wang et al. 2022). Although LSU and ITS rDNA sequences are widely used for species identification within *Coniochaeta*, these markers are insufficient to resolve species boundaries and relationships among closely related taxa (Perdomo et al. 2013; Nasr et al. 2018; Harrington et al. 2019; Arnold et al. 2021a, 2021b; Campos-Quiroz et al. 2023). Arnold et al. (2021a, 2021b) further demonstrated that species complexes in *Coniochaeta* cannot be resolved using ITS alone and that multilocus data from protein-coding genes, such as actin (*act*), the second-largest subunit of RNA polymerase II (*rpb2*), and translation elongation factor 1-alpha (*tef1-α*), are required to clarify phylogenetic relationships.

Members of *Coniochaeta* occur as saprobes, endophytes, and plant pathogens and have a worldwide distribution across various substrates, including animal dung, butter, leaf litter, lichen, plants, rock, soil, water, and wood in terrestrial and freshwater habitats and mangrove sediments (Damm et al. 2010; Raja et al. 2012; Zhang et al. 2016; Harrington et al. 2019; Arnold et al. 2021a, 2021b; Si et al. 2021; Li et al. 2023; Silva et al. 2023; Czachura 2024; Sastoque et al. 2024). Additionally, some species, including *C. hoffmannii*, *C. massiliensis*, and *C. mutabilis*, have been reported as causal agents of human and animal infections, including fungal keratitis, endophthalmitis, peritonitis, septic shock, sinusitis, and canine osteomyelitis (Ahmad et al. 1985; Marriott et al. 1997; Sakaeyama et al. 2007; Taniguchi et al. 2009; Kabtani et al. 2022; Rajan et al. 2023; Pegany et al. 2024). *Coniochaeta* species, particularly endophytes, are a source of bioactive secondary metabolites and exhibit antibacterial, antifungal, and antioxidative activities (Asgari et al. 2007; Rosa et al. 2013; Xie et al. 2015; Fu et al. 2021; El-Sayed et al. 2025). Moreover, some *Coniochaeta* species, e.g., *C. ligniaria* and *C. velutina*, also exhibit growth-promoting activity in certain plants and inhibit phytopathogens (Kokaew et al. 2011; Rosa et al. 2013; Chen et al. 2022; Shah et al. 2022; Wang et al. 2022; Zheng et al. 2026).

Thailand, located in a tropical region with diverse ecosystems and climates, has been identified by numerous mycologists as a hotspot for fungal diversity and the discovery of new fungal taxa (Hyde et al. 2018; Preedanon et al. 2023; Suetrong et al. 2023; Suwannarach et al. 2023, 2024; Kodchasee et al. 2026; Zhang et al. 2026). As part of a survey of microfungi from rock and soil samples in northern Thailand during 2024–2025, six strains of *Coniochaeta* were isolated. Therefore, the objective of this study is to characterize and accurately identify these six fungal strains. Based on morphological characteristics and molecular evidence, two new species are described here as *Coniochaeta saxicola* and *C. terricola*. These taxa are illustrated and described in detail based on morphological characteristics and multilocus phylogenetic analyses.

## Materials and methods

### Sample collection and isolation of fungi

Rock and soil samples were collected from Lampang and Lamphun Provinces, respectively, in northern Thailand during 2024–2025. The samples were placed in separate zip-lock plastic bags, kept in an ice box, and transported to the microbiological laboratory at the Center of Excellence in Microbial Diversity and Sustainable Utilization, Chiang Mai University, Chiang Mai, Thailand. For fungal isolation, 1 g of each sample was added to 9 mL of 0.85% (w/v) sodium chloride (NaCl) solution in a 150 mL Erlenmeyer flask and thoroughly mixed using a vortex mixer. The resulting suspension was subjected to a series of 10-fold dilutions. A 100 µL aliquot from each dilution was spread onto dichloran rose bengal chloramphenicol (DRBC) agar. The plates were incubated at 25 °C for 1 week and monitored every 48 h for fungal growth. Hyphal tips from individual colonies were transferred to fresh potato dextrose agar (PDA; Difco, Becton, Dickinson and Company, USA) and incubated at 25 °C in the dark to obtain pure cultures. The living pure cultures were stored in 20% glycerol in screw-cap tubes at −80 °C for long-term preservation. Dried cultures and pure cultures were deposited in the Herbarium of the Department of Biology (CMUB) and the Culture Collection of the Sustainable Development of Biological Resources Laboratory (SDBR), Faculty of Science, Chiang Mai University, Thailand, respectively. Nomenclatural and taxonomic information was registered in the MycoBank database (MycoBank 2026).

### Morphological studies

Mycelial plugs (5 mm in diameter), cut from the leading edges of 7-day-old PDA cultures of each fungal strain, were transferred onto malt extract agar (MEA; Gibco, Life Technologies Corporation, USA), oatmeal agar (OA; Difco, Becton, Dickinson and Company, USA), synthetic nutrient-deficient agar (SNA; 1 g/L KH_2_PO_4_, 1 g/L KNO_3_, 0.5 g/L MgSO_4_.7H_2_O, 0.5 g/L KCl, 0.2 g/L sucrose, 0.5 g/L glucose, and 20 g/L agar), and PDA to observe colony characteristics and sporulation after 2 weeks of incubation at 25 °C in the dark. Moreover, the cardinal growth temperatures (4, 10, 15, 20, 25, 30, 37, and 45 °C) were determined on MEA and PDA. Macroscopic characteristics were examined using a Motic SMZ 168 Series microscope, and images were captured using a Carl Zeiss GmbH stereomicroscope equipped with an AxioCam ERC 5S camera. Microscopic structures were mounted in distilled water, and images were captured using a Nikon ECLIPSE Ni light microscope (Tokyo, Japan) connected to a Nikon DS-Ri2 camera. All measurements were taken using Tarosoft® Image Framework software, with at least 30 measurements taken for each structure. Images used in the figures were processed using Adobe Photoshop version 22.4.2 (Adobe Systems U.S.A.).

### DNA extractions, PCR amplification, and sequencing

Fungal genomic DNA was extracted from 2-week-old mycelium grown on PDA using the Fungal DNA Extraction Kit (FAVORGEN, Ping-Tung, China), following the manufacturer’s protocol. The primer pairs ITS5/ITS4 (White et al. 1990), LR0R/LR5 (Vilgalys and Hester 1990), EF1-983F/EF1-2218R (Rehner and Buckley 2005), Bt2a/Bt2b (Glass and Donaldson 1995), RPB1-Af/RPB1-Cr (Stiller and Hall 1997; Matheny et al. 2002), and fRPB2-5F/fRPB2-7cR (Liu et al. 1999) were used for amplification of ITS, LSU, *tef1-α*, beta-tubulin (*tub*), the largest subunit of RNA polymerase II (*rpb1*), and *rpb2*, respectively. PCR amplification was performed as described by Suwannarach et al. (2024). The PCR thermal cycling conditions were as follows: initial denaturation at 95 °C for 5 min, followed by 40 cycles of denaturation at 95 °C for 30 s, annealing at the specific temperatures (56 °C for ITS, *rpb1*, *tef1-α*, and *tub* and 52 °C for LSU and *rpb2*) for 30 s, elongation at 72 °C for 1 min, and a final extension at 72 °C for 10 min. PCR products were inspected by electrophoresis on 1% agarose gels under UV light. PCR amplicons were purified using the NucleoSpin® Gel and PCR Clean-up Kit (Macherey-Nagel, Germany), following the manufacturer’s protocol, and submitted to 1^st^ BASE Company (Kembangan, Malaysia) for sequencing. Raw sequences were examined and assembled using SeqMan™ II version 5.00 (DNASTAR, Madison, WI, USA). The newly generated sequences were deposited in GenBank.

### Phylogenetic analyses

The closest matching taxa were determined using nucleotide BLAST searches of the NCBI nucleotide database (http://blast.ncbi.nlm.nih.gov/). Due to the limited availability of comparable sequences for *rpb1*, *rpb2*, and *tub*, these loci were not included in the phylogenetic analyses. In addition, pairwise sequence similarity between two specific sequences was further assessed using the NCBI BLAST 2 Sequences (BLAST2Seq) tool. The LSU, ITS, and *tef1-α* sequences used for the analyses were retrieved from previous studies (Campos-Quiroz et al. 2023; Li et al. 2023; Silva et al. 2023; Crous et al. 2025) and the GenBank database and are listed in Table 1. *Cordana solitaria* (CBS 214.86) and *Co. verruculosa* (CBS 121870) were selected as the outgroup taxa based on phylogenetic analyses. All sequences were aligned using the MAFFT sequence alignment server (Katoh et al. 2019; http://mafft.cbrc.jp/alignment/server/) and checked manually using BioEdit v. 7.0.9.1 (Hall 1999). The phylogenetic tree based on the combined LSU, ITS, and *tef1-α* sequence dataset was analyzed using maximum likelihood (ML) and Bayesian inference (BI) methods.

**Table 1.** Taxa included in the phylogenetic analyses and their corresponding collection numbers and GenBank accession numbers. Ex-type strains are indicated by a superscript “^T^” following the collection numbers. “–” indicates that sequence data were unavailable in GenBank.

**Fungal species**	**Collection number**	**GenBank accession number**			**Reference**
		**LSU**	**ITS**	***tef1-α***	
*Coniochaeta acaciae*	MFLUCC 17-2298^T^	MG062737	MG062735	–	Samarakoon et al. 2018
*C. acaciae*	CX37	MW750757	MW750761	–	Si et al. 2021
*C. africana*	CBS 120868^T^	GQ154601	GQ154539	–	Damm et al. 2010
*C. ambigua*	PAD S00027^T^	–	ITS1: MW626895	–	Forin et al. 2021
			ITS2: MW626903		
*C. angustispora*	CBS 144.70	MH871308	MH859528	–	Vu et al. 2019
*C. arenariae*	MFLUCC 18-0405^T^	MN017893	–	–	Dayarathne et al. 2020
*C. arenariae*	MFLUCC 18-0409	MN017896	MN047126	–	Dayarathne et al. 2020
*C. arxii*	CBS 192.89	–	MH862164	–	Vu et al. 2019
*C. aurantiaca*	CGMCC 3.22339^T^	OQ758133	OQ798936	–	Li et al. 2023
*C. aurantiaca*	CGMCC 3.22340	OQ758134	OQ798937	–	Li et al. 2023
*C. australiensis*	BRIP 74375a^T^	OR670002	NR_191317	–	Tan 2023
*C. baysunika*	MFLUCC 17-0830^T^	MG828996	MG828880	MG829196	Wanasinghe et al. 2018
*C. boothii*	CBS 381.74^T^	AJ875226	NR_159776	–	García et al. 2006; Vu et al. 2019
*C. canina*	UTHSC 11-2460^T^	JX481774	JX481775	–	Troy et al. 2013
*C. caraganae*	MFLUCC 18-0780^T^	MT573223	MT573224	–	Jayawardena et al. 2022
*C. cateniformis*	UTHSC 01-1644^T^	HE610329	HE610331	–	Perdomo et al. 2013
*C. cephalothecoides*	L821	KY064030	KY064029	–	Han et al. 2017
*C. chordicola*	PAD S00030^T^	–	ITS1: MW626898	–	Forin et al. 2021
			ITS2: MW626905		
*C. cipronana*	CBS 144016^T^	–	MF422164	–	Coronado-Ruiz et al. 2018
*C. coluteae*	MFLUCC 17-2299^T^	MG137252	MG137251	MG194425	Samarakoon et al. 2018
*C. corticalis*	JKI-GP-23-050^T^	PV259250	PV272673	PV287720	Crous et al. 2025
*C. cruciata*	FMR 7409	AJ875222	–	–	García et al. 2006
*C. cymbiformispora*	NBRC 32199^T^	LC146726	LC146726	–	Unpublished
*C. cypraeaespora*	CBS 365.93^T^	MH874073	MH862420	–	Vu et al. 2019
*C. dakotensis*	PAD S00035	–	ITS1: MW626902	–	Forin et al. 2021
			ITS2: MW626910		
*C. deborreae*	CBS 147215^T^	MW883808	MW883413	MW890106	Crous et al. 2021
*C. decumbens*	CBS 153.42^T^	AF353597	HE610337	–	Weber et al. 2002; Perdomo et al. 2013
*C. dendrobiicola*	DLCCR7^T^	MK225603	MK225602	–	Crous et al. 2019
*C. discoidea*	CBS 158.80^T^	NG_064120	NR_159779	–	García et al. 2006
*C. discoidea*	SANK12878	AY346297	–	–	Huhndorf et al. 2004
*C. discospora*	CBS 168.58	MH869278	MH857740	–	Vu et al. 2019
*C. elegans*	FF0093	–	MZ262389	MZ267815	Arnold et al. 2021b
*C. ellipsoidea*	CBS 137.68^T^	MH870804	MH859091	–	Vu et al. 2019
*C. endophytica*	AEA 9094^T^	EF420069	EF420005	MK693159	Harrington et al. 2019
*C. euphorbiae*	CBS 139768^T^	KP941075	KP941076	–	Nasr et al. 2018
*C. extramundana*	CBS 247.77^T^	MH872828	MH861057	–	Vu et al. 2019
*C. fasciculata*	CBS 205.38^T^	AF353598	HE610336	MK693152	Weber et al. 2002; Perdomo et al. 2013; Harrington et al. 2019
*C. fermentaria*	JKI-GP-22-032^T^	PV259253	PV272676	PV287723	Crous et al. 2025
*C. fibricola*	JKI-GP-22-031^T^	PV259251	PV272674	PV287721	Crous et al. 2025
*C. fibrosae*	CGMCC3.20304^T^	MW750758	MW750760	–	Si et al. 2021
*C. fibrosae*	CX04D1	MW750755	MW750756	–	Si et al. 2021
*C. fodinicola*	CBS 136963^T^	KF857172	JQ904603	–	Vázquez-Campos et al. 2014
*C. fragariicola*	CBS 149284^T^	OP962069	OP962067	OP973030	Campos-Quiroz et al. 2023
*C. geophila*	PAD S00031^T^	–	ITS2: MW626906	–	Forin et al. 2021
*C. gigantospora*	ILLS 60816^T^	JN684909	JN684909	–	Raja et al. 2012
*C. groatii*	BRIP 74441a^T^	–	OQ297064	–	Tan et al. 2023
*C. hansenii*	CBS 885.68	AJ875223	OP962072	–	García et al. 2006; Campos-Quiroz et al. 2023
*C. hansenii*	RGM 3311	OP962070	OP962068	–	Campos-Quiroz et al. 2023
*C. hoffmannii*	CBS 245.38^T^	AF353599	MG491499	MK693150	Weber et al. 2002; Leonhardt et al. 2018; Harrington et al. 2019
*C. hoffmannii*	DSM 2693	OM366270	OM366155	OM640095	Kabtani et al. 2022
*C. iranica*	CBS 139767^T^	KP941077	KP941078	–	Nasr et al. 2018
*C. krabiensis*	MFLU 16-1230^T^	MN017892	–	–	Dayarathne et al. 2020
*C. leucoplaca*	CBS 486.73	MH872465	OP962071	–	Vu et al. 2019; Campos-Quiroz et al. 2023
*C. ligniaria*	CBS 424.65	MH870292	MH858650	–	Vu et al. 2019
*C. lignicola*	CBS 267.33^T^	AF353601	HE610335	MK693154	Weber et al. 2002; Perdomo et al. 2013; Harrington et al. 2019
*C. lutea*	CBS 121445^T^	GQ154603	GQ154541	MK693161	Damm et al. 2010; Harrington et al. 2019
*C. luteorubra*	FeC127	–	MW447035	–	Bilański and Kowalski 2022
*C. luteorubra*	FMR 10721^T^	HE610328	HE610330	–	Perdomo et al. 2013
*C. luteoviridis*	CBS 206.38^T^	AF353603	HE610333	–	Weber et al. 2002; Perdomo et al. 2013
*C. malacotricha*	F2105	AF353588	–	–	Weber et al. 2002
*C. malacotricha*	F2106	AF353589	–	–	Weber et al. 2002
*C. marina*	MFLUCC 18-0408^T^	MK458765	MK458764	–	Jones et al. 2020
*C. massiliensis*	PMML0158^T^	OM366268	OM366153	OM640093	Kabtani et al. 2022
*C. mongoliae*	CGMCC3.20250^T^	MW077646	MW077645	–	Si et al. 2021
*C. montana*	SR0076^T^	–	MZ262414	MZ267840	Arnold et al. 2021b
*C. monsterae*	URM 8284^T^	MZ648891	MZ648895	–	Silva et al. 2023
*C. monsterae*	URM 8655	MZ648892	MZ648896	–	Silva et al. 2023
*C. mutabilis*	CBS 157.44^T^	AF353604	HE610334	–	Weber et al. 2002; Perdomo et al. 2013
*C. navarrae*	CBS 141016^T^	KU762326	KU762326	KU762332	Friebes et al. 2016
*C. nepalica*	NBRC 30584^T^	LC146727	LC146727	–	Unpublished
*C. nivea*	AK0926^T^	–	MZ262367	MZ267793	Arnold et al. 2021b
*C. notelaeae*	BRIP 66975a^T^	–	PV074481	–	Tan et al. 2025b
*C. ornata*	FMR 7415^T^	AJ875228	–	–	García et al. 2006
*C. ostrea*	CBS 507.70^T^	NG_064080	NR_159772	–	Vu et al. 2019
*C. palaoa*	NC0604^T^	–	MZ241149	MZ241188	Arnold et al. 2021a
*C. polymorpha*	CBS 132722^T^	HE863327	HE863327	–	Khan et al. 2013
*C. polysperma*	CBS 669.77^T^	MH872868	MH861109	–	Vu et al. 2019
*C. prunicola*	CBS 120875^T^	GQ154602	GQ154540	MK693162	Damm et al. 2010; Harrington et al. 2019
*C. psammospora*	CBS 590.63^T^	OQ055448	OQ429536	–	Hou et al. 2023
*C. pulveracea*	CBS 114628	GQ351560	MW883414	–	van Heerden et al. 2011; Crous et al. 2021
*C. punctulata*	CBS 159.80	MH873024	MH861254	–	Vu et al. 2019
*C. queenslandica*	BRIP 74376a^T^	–	NR_191318	–	Tan 2023
*C. rankiniae*	BRIP 74950a^T^	OR122292	OR122281	–	Tan and Shivas 2023
*C. rhopalochaeta*	CBS 109872	GQ351561	NR_172554	–	van Heerden et al. 2011; Crous et al. 2021
*C. riskalishoyakubovii*	TASM 6166^T^	OQ355012	OQ355014	OQ354721	Senanayake et al. 2023
*C. rosae*	TASM 6127^T^	MG828999	MG828883	MG829197	Wanasinghe et al. 2018
*C. savoryi*	CBS 725.74^T^	MH872627	MH860890	–	Vu et al. 2019
***C. saxicola***	**SDBR-CMU525^T^**	**PZ298080**	**PZ298086**	**PZ309319**	**This study**
***C. saxicola***	**SDBR-CMU526**	**PZ298081**	**PZ298087**	**PZ309320**	**This study**
***C. saxicola***	**SDBR-CMU896**	**PZ298082**	**PZ298088**	**PZ309321**	**This study**
*C. simbalensis*	NFCCI 4236^T^	MG917738	MG825743	–	Unpublished
*C. sinensis*	CGMCC3.20306^T^	MW422265	MW422269	–	Si et al. 2021
*C. sordaria*	CBS 492.73	MH878380	–	–	Vu et al. 2019
*C. subcorticalis*	CBS 551.75	AF353593	MW883416	–	Weber et al. 2002; Crous et al. 2021
*C. taeniospora*	CBS 141014^T^	KU762324	KU762324	–	Friebes et al. 2016
*C. taeniospora*	MFLU 17-0832	MN336234	MN337230	–	Unpublished
*C. teitelbaumiae*	BRIP 72364b^T^	PQ882520	PQ882520	–	Tan et al. 2025a
***C. terricola***	**SDBR-CMU889^T^**	**PZ298083**	**PZ298089**	**PZ309322**	**This study**
***C. terricola***	**SDBR-CMU890**	**PZ298084**	**PZ298090**	**PZ309323**	**This study**
***C. terricola***	**SDBR-CMU891**	**PZ298085**	**PZ298091**	**PZ309324**	**This study**
*C. tetraspora*	CBS 139.68	MH870806	MH859093	–	Vu et al. 2019
*C. tritici*	IRAN 3464C	MW432524	MW432175	–	Crous et al. 2021
*C. velutina*	CBS 121444	GQ154605	GQ154544	–	Damm et al. 2010
*C. velutina*	CBS 981.68	MH870991	MH859264	–	Vu et al. 2019
*C. velutinosa*	CBS117678^T^	GU553330	GU553327	–	Zare et al. 2010
*C. velutinosa*	CGMCC3.20249	MW346687	MW298866	–	Si et al. 2021
*C. verticillata*	CBS 816.71^T^	AJ875232	MH860369	–	García et al. 2006; Vu et al. 2019
*C. vineae*	KUMCC 17-0322^T^	MN473512	MN473469	–	Hyde et al. 2020
*C. weberae*	JKI-GP-23-051^T^	PV259252	PV272675	PV287722	Crous et al. 2025
*Cordana verruculosa*	CBS 121870	HE672163	HE672152	–	Hernández-Restrepo et al. 2014
*Co. solitaria*	CBS 214.86^T^	HE672161	HE672150	–	Hernández-Restrepo et al. 2014

The ML analysis was run using RAxML-HPC2 on ACCESS (8.2.12) (Stamatakis 2014) through the CIPRES web portal (Miller et al. 2010), with 1,000 rapid bootstrap (BS) replicates under the GTRGAMMA nucleotide substitution model. The BI analysis was conducted using Markov chain Monte Carlo (MCMC) sampling in MrBayes v. 3.2.6 (Ronquist et al. 2012). Using MrModeltest v. 2.3 (Nylander 2008) with the Akaike information criterion (AIC), the GTR+I+G substitution model was determined to be the best-fit model for the sequence evolution of LSU, ITS, and *tef1-α*. Four simultaneous Markov chains were set to run for 16 million generations, and trees were sampled every 100^th^ generation. When the standard deviation of split frequencies was less than 0.01, the run was terminated, yielding a total of 160,000 trees. The first 40,000 trees were discarded as the burn-in phase, and the remaining trees were used to estimate posterior probabilities (PP) for the majority-rule consensus tree. Phylogenetic trees were drawn using FigTree v. 1.4.0 (Rambaut 2016) and modified using Adobe Illustrator version 25.2.3 (Adobe Systems, USA).

## Results

### Fungal isolation

In this study, six fungal strains were deposited in the SDBR Culture Collection under the accession numbers SDBR-CMU525, SDBR-CMU526, SDBR-CMU889, SDBR-CMU890, SDBR-CMU891, and SDBR-CMU896. Based on morphological characteristics, all fungal strains were assigned to *Coniochaeta*. Subsequently, a multilocus phylogenetic approach was employed to confirm their phylogenetic placement.

### Phylogenetic analyses

**Figure 1.**
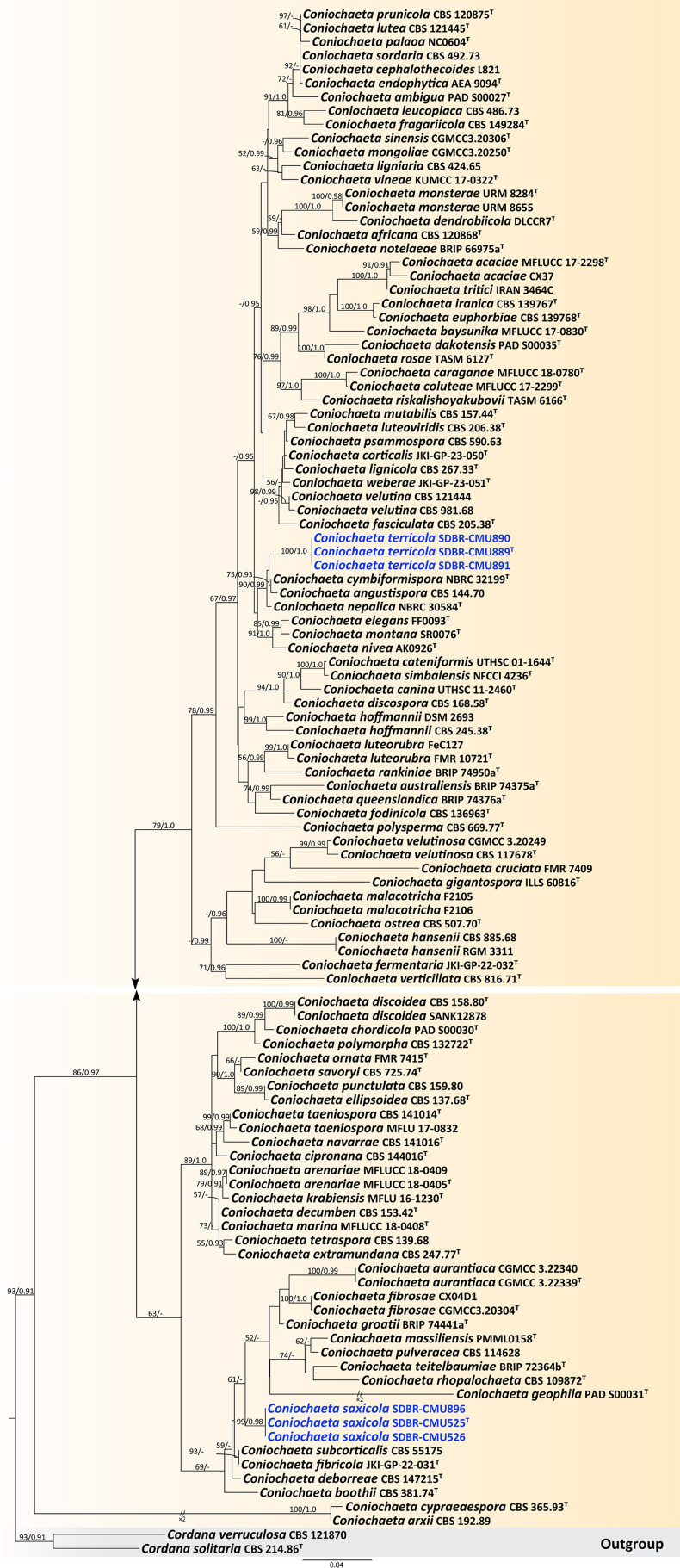
Phylogenetic tree generated from maximum likelihood analysis of a combined dataset of LSU, ITS, and *tef1-α* sequence data from 111 fungal strains. *Cordana solitaria* CBS 214.86 and *Co. verruculosa* CBS 121870 were used as the outgroup taxa. Bootstrap support values for maximum likelihood (ML) equal to or greater than 50% and Bayesian posterior probabilities (PP) equal to or greater than 0.90 are indicated at the branches as ML/BI. Ex-type strains are denoted by a superscript “^T^” after the accession number. Newly sequenced taxa in this study are shown in blue.

The combined LSU, ITS, and *tef1-α* sequence analyses included 111 strains, comprising six newly sequenced strains (SDBR-CMU525, SDBR-CMU526, SDBR-CMU889, SDBR-CMU890, SDBR-CMU891, and SDBR-CMU896) and two outgroup taxa [*Cordana solitaria* (CBS 214.86) and *Co. verruculosa* (CBS 121870)]. The combined dataset comprised 2,354 characters (LSU: 1–881, ITS: 882–1,482, and *tef1-α*: 1,483–2,354), including gaps. Single-gene analyses were also conducted, and their taxonomic and topological stability was compared with that of the combined-gene analyses. RAxML analysis of the combined dataset, with a final ML optimization likelihood value of −15115.125789, contained 956 distinct alignment patterns and 47.17% undetermined characters or gaps. The estimated base frequencies were as follows: A = 0.240932, C = 0.257684, G = 0.282317, and T = 0.219066; substitution rates were AC = 1.503391, AG = 2.007986, AT = 1.314680, CG = 1.440351, CT = 7.112900, and GT = 1.000000; and the gamma distribution shape parameter was *α* = 0.475190. Bayesian posterior probabilities (PP) from MCMC were evaluated using a final average standard deviation of split frequencies of 0.008762. The topologies of the trees generated from the ML and BI analyses were similar. Therefore, the phylogenetic tree generated from the ML analysis is shown in Fig. 1. The results indicated that the six fungal strains in this study formed two distinct monophyletic lineages within the genus *Coniochaeta*, with high BS and PP support values. Three strains (SDBR-CMU525, SDBR-CMU526, and SDBR-CMU896) are described here as *C. saxicola*, whereas three strains (SDBR-CMU889, SDBR-CMU890, and SDBR-CMU891) are introduced as *C. terricola*. *Coniochaeta saxicola* formed a sister clade to *C. aurantiaca*, *C. fibrosae*, *C. groatii*, *C. massiliensis*, *C. pulveracea*, *C. teitelbaumiae*, *C. rhopalochaeta*, *C. geophila*, *C. subcorticalis*, *C. fibricola*, *C. deborreae*, and *C. boothii*. In contrast, *C. terricola* formed a sister clade to *C. cymbiformispora* and *C. angustispora*.

### Taxonomic descriptions

#### Coniochaeta saxicola

Taxon classificationFungiConiochaetalesConiochaetaceae

N. Suwannar., J. Kumla & Senwanna sp. nov.

E449E009-9967-5C37-B44F-59278CDEC835

863211

Fig. 2

##### Etymology.

The name “*saxicola*” refers to rock inhabitants.

**Figure 2.**
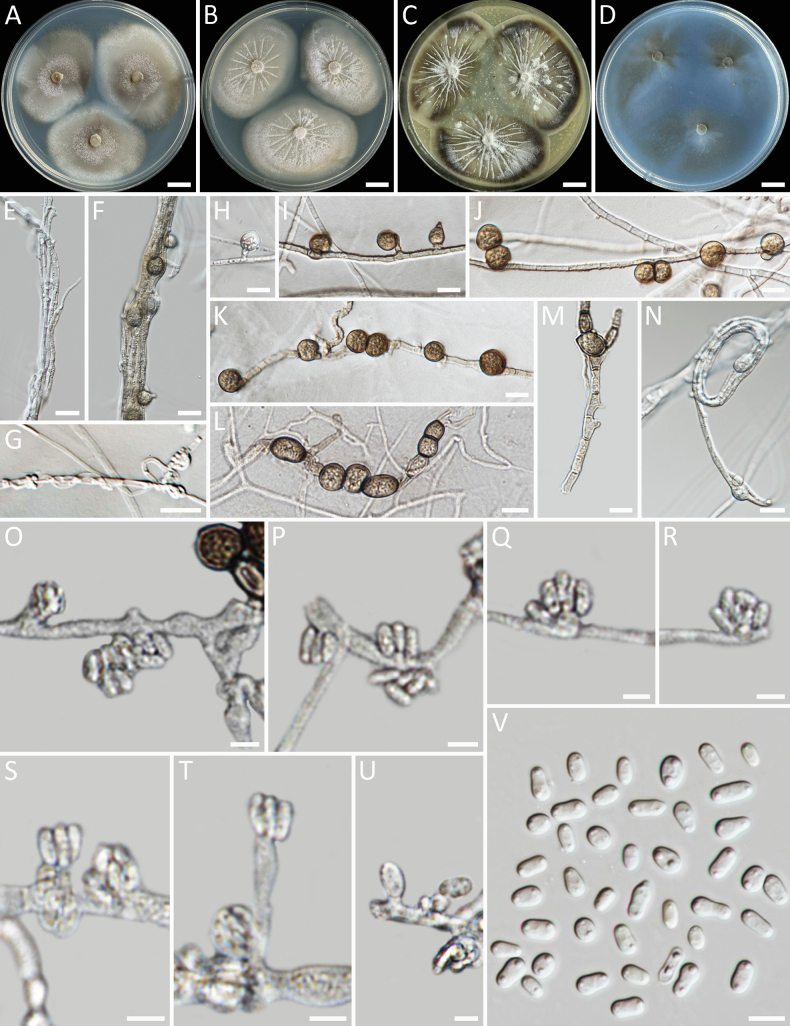
*Coniochaeta saxicola* (SDBR-CMU525, ex-type). **A–D**. Colonies on PDA, MEA, OA, and SNA, respectively, after 14 d at 25 °C; **E**. Immature packed hyphae; **F**. Mature packed hyphae mixed with chlamydospores; **G**. Spirally intertwined hyphae; **H–M**. Young and mature chlamydospores; **N** Hyphal coil and conidiophores; **O–U**. Conidiogenous cells on hyphal cells; **V**. Conidia. Scale bars: 10 mm (**A–D**), 10 µm (**E–N**), 5 µm (**O–V**).

##### Type.

Thailand • Lamphun Province, Mueang Lamphun District, Sribuaban Subdistrict, Chiang Mai University Hariphunchai Campus, 18°32'11"N, 99°07'30"E, elevation 354 m, isolated as a rock-inhibiting fungus from rock samples, 28 June 2024, N. Suwannarach, holotype, CMUB40148 (a dried culture grown on PDA), ex-type living culture SDBR-CMU525.

##### Cultural characteristics.

Colonies on different agar media were incubated in the dark at 25 °C for 14 days. On PDA, colonies 48 to 55 mm in diameter, flat, velvety, with an entire edge, light grey mixed with light brownish grey at center, paler at the margin; reverse light grey. On MEA, colonies 54 to 57 mm in diameter, light grey to light brownish grey at center, effuse, with rough surface towards center, radial grooves, diffuse margin with light grey to white; reverse light grey at center, with light grey at margin. On OA, colonies 55 to 60 mm in diameter, dark greyish brown with white at center, effuse, with rough surface towards center, radial grooves, diffuse margin with dark greyish brown and creamy white; reverse greyish brown at center with dark greyish brown and creamy white at margin. On SNA, colonies 59 to 65 mm in diameter, dark greyish brown with white at center, immersed mycelia, effuse; reverse same color scheme. ***Sexual morph*** Undetermined. ***Asexual morph in vitro*** hyphomycetous. ***Vegetative hyphae*** 2.0–3.8(–4.7) μm wide, hyaline to light brown, septate, branched, thin- and smooth-walled, tightly packed, occasionally forming characteristic hyphal coils and spirally intertwined hyphal filaments. ***Conidiophores*** hyaline, micronematous, undifferentiated. ***Conidiogenous cells*** phialidic, ampulliform, single or in clusters on short lateral branches, hyaline, 2.6–5.3 × 2.4–4.6 μm (x̄ = 3.7 × 3.6 μm, *n* = 30), without the formation of a collarette. ***Conidia*** oblong-ellipsoidal to ovoid, aseptate, hyaline, smooth-walled, round at both ends, 2.7–5.5 × 1.9–3.4 μm (x̄ = 4.6 × 2.6 μm, *n* = 60, *L/W* ratio = 1.7). ***Chlamydospores*** solitary or in short chains, mostly intercalary, globose to subglobose, hyaline when immature, brown to dark brown at maturity, (7.5–)9–25(–32) × (4.8–)7.2–11.2(–13) μm (x̄ = 16.1 × 9.4 μm, *n* = 50).

##### Cardinal temperatures for growth on MEA and PDA after two weeks.

Minimum at 4 °C (5 to 7 mm in diameter). 10 °C (27 to 38 mm in diameter), 15 °C (33 to 49 mm in diameter), 20 °C (35 to 50 mm in diameter), 25 °C (48 to 58 mm in diameter). Optimum and maximum at 30 °C (65 to 72 mm in diameter). No growth was observed at 37 °C and 45 °C.

##### Additional strains examined.

Thailand • Lamphun Province, Mueang Lamphun District, Sribuaban Subdistrict, Chiang Mai University Hariphunchai Campus, 18°32'11"N, 99°07'30"E, elevation 354 m, isolated as a rock-inhibiting fungus from rock samples, 28 June 2024, N. Suwannarach, living culture SDBR-CMU526 and SDBR-CMU896.

##### Additional GenBank numbers.

SDBR-CMU525: PZ309325 (*tub*), PZ309313 (*rpb1*), and PZ309316 (*rpb2*); SDBR-CMU526: PZ309326 (*tub*), PZ309314 (*rpb1*), and PZ309317 (*rpb2*); SDBR-CMU896: PZ309327 (*tub*), PZ309315 (*rpb1*), PZ309318 (*rpb2*).

##### Notes.

In nucleotide BLAST searches, the ITS, LSU, and *tef1-α* sequences of all three strains of *C. saxicola* (SDBR-CMU525, SDBR-CMU526, and SDBR-CMU896) showed the closest match to *C. deborreae* BE19_001008 (ex-type), with similarities of 96.73%, 99.39%, and 97.55%, respectively. Multilocus phylogenetic analyses based on a concatenated LSU, ITS, and *tef1-α* sequence dataset showed that the three strains of *C. saxicola* formed a distinct monophyletic clade, clustering with *C. aurantiaca*, *C. boothii*, *C. fibricola*, *C. fibrosae*, *C. deborreae*, *C. geophila*, *C. groatii*, *C. massiliensis*, *C. pulveracea*, *C. rhopalochaeta*, *C. subcorticalis*, and *C. teitelbaumiae*. Comparative analyses of LSU, ITS, and *tef1-α* sequences among phylogenetically related taxa revealed that *C. saxicola* was distinct from the other taxa (Table 2). The morphological distinctness of *C. saxicola* from other phylogenetically related taxa is presented in Table 3. The conidia of *C. saxicola* are smaller than those of *C. massiliensis* (Kabtani et al. 2022) and *C. rhopalochaeta* (Romero et al. 1999) but larger than those of *C. deborreae* (Crous et al. 2021) and *C. fibricola* (Crous et al. 2025). The two conidial types of *C. fibrosae* (Si et al. 2021) are distinct from those of *C. saxicola*. The smaller conidiogenous cells and lack of a collarette in *C. saxicola* distinguish it from *C. aurantiaca* (Li et al. 2023). Additionally, the presence of chlamydospores in *C. saxicola* distinguishes it from *C. fibricola* (Crous et al. 2025), *C. fibrosae* (Si et al. 2021), and *C. massiliensis* (Kabtani et al. 2022). However, morphological comparisons of *C. saxicola* with *C. boothii* (Manoharachary and Rao 1973), *C. geophila* (Forin et al. 2021), *C. groatii* (Tan et al. 2023), *C. pulveracea* (Munk 1948), *C. subcorticalis* (Matsushima 1971), and *C. teitelbaumiae* (Tan et al. 2025a) could not be performed because of the lack of available asexual morph descriptions, which requires further study. Thus, *C. saxicola* is introduced as a novel species.

**Table 2.** Comparison of nucleotide polymorphisms in the ITS, LSU, and *tef1-α* regions between *Coniochaeta saxicola* (SDBR-CMU525) and other phylogenetically related species.

***Coniochaeta* species**	**Strain numbers**	**ITS**	**LSU**	***tef1-α***	**References**
*C. aurantiaca*	CGMCC 3.22339	7.25% (37/510 bp)	2.68% (22/822 bp)	–	Li et al. 2023
*C. boothii*	CBS 381.74	4.90% (24/490 bp)	1.53% (7/456 bp)	–	Vu et al. 2019; García et al. 2006
*C. deborreae*	CBS 147215	2.88% (16/555 bp)	0.61% (5/814 bp)	2.45% (22/898 bp)	Crous et al. 2021
*C. fibricola*	JKI-GP-22-031	2.68% (15/560 bp)	0.56% (5/883 bp)	–	Crous et al. 2025
*C. fibrosae*	CGMCC3.20304	6.69% (37/553 bp)	2.30% (18/781 bp)	–	Si et al. 2021
*C. geophila*	PAD S00031 (only ITS2)	17.18% (28/163 bp)	–	–	Forin et al. 2021
*C. groatii*	BRIP74441	4.05% (23/568 bp)	–	–	Tan et al. 2023
*C. massiliensis*	PMML0158	6.94% (22/317 bp)	2.91% (8/275 bp)	3.59% (16/446 bp)	Kabtani et al. 2022
*C. pulveracea*	CBS 114628	8.69% (47/541 bp)	4.26% (26/510 bp)	–	van Heerden et al. 2011; Crous et al. 2021
*C. rhopalochaeta*	CBS 109872	9.47% (54/570 bp)	4.12% (21/510 bp)	–	van Heerden et al. 2011
*C. subcorticalis*	CBS 551.75	2.69% (15/557 bp)	1.93% (9/467 bp)	–	Weber et al. 2002; van Heerden et al. 2011; Crous et al. 2021
*C. taeniospora*	CBS 141014	7.90% (46/580 bp)	2.87% (25/870 bp)	–	Friebes et al. 2016
*C. teitelbaumiae*	BRIP 72364b	8.97% (47/524 bp)	3.35% (19/567 bp)	–	Tan et al. 2025a

**Table 3.** Comparison of asexual morph characteristics between *Coniochaeta saxicola* and other phylogenetically related species.

***Coniochaeta* species**	**Microscopic structures**			**Reference**
	**Conidiogenous cells**	**Conidia**	**Chlamydospores**	
*C. aurantiaca*	Enteroblastic, sometimes inconspicuous, 4.5–17.5 × 1.5–2.5 μm, flared collarettes present or absent, when present 0.5–2.5 × 0.5–1.0 μm	Mostly intercalary and terminal, few lateral, variable in size and shape, mostly subglobose, ellipsoidal to ovoid, unicellular, smooth, hyaline, 2.0–5.0 × 1.5–4.0 μm	Not reported	Li et al. 2023
*C. deborreae*	Terminal, lateral or intercalary, mono- or polyphialidic, cylindrical, ampulliform or ventricose, nearly globose, 4–11 × 2–3.5 μm, hyaline, with conspicuous periclinal thickening and distinguishable, usually cylindrical collarettes, 1–2 × 1–1.5 μm	Ellipsoidal to oblong, with rounded apex and truncate base, aseptate, hyaline, smooth-walled, 2.5–4 × 1.5–2 μm	Solitary or in short chains, subglobose, pale brown, 6.5–13 × 5–9 μm	Crous et al. 2021
*C. fibricola*	Formed directly on hyphae, mostly reduced to protrusions	Solitary, ellipsoidal, partly arched, apex obtuse, aseptate, hyaline, smooth-walled, 2.7–4.8 × 2.2–3.3 µm	Absent	Crous et al. 2025
*C. fibrosae*	Phialidic or oval in shape, single or in clusters on short lateral branches, hyaline, 2.9–7.2 × 1.8–3.7 μm	Two types of conidia were observed: dumb-bell-shaped, hyaline, one-celled, with hyphae emerging from both ends, 7.6–16.5 × 2.3–4.1; oblong conidia, hyaline, one-celled, often oblong to ellipsoidal in shape, 3.4–6.8 × 1.4–2.7 μm	Absent	Si et al. 2021
*C. massiliensis*	Cylindrical adelophialides (short phialides without septum), discrete phialides with conical tips	Ellipsoidal to cylindrical and rarely curved conidia, and nonseptate with thin and smooth conidial walls, 6–10 × 2–3 µm	Absent	Kabtani et al. 2022
*C. rhopalochaeta*	Enteroblastic (phialidic), intercalary or discrete with narrow apices, inconspicuously flared collarettes, 10–18 × 3.5–5 µm	Oblong-ellipsoid to cylindric, 5–8 × 3–5 µm	Terminal or intercalary, in short chains	Romero et al. 1999
*C. saxicola*	Phialidic, ampulliform, single or in clusters on short lateral branches, hyaline, 2.6–5.3 × 2.4–4.6 μm, without the formation of a collarette	Oblong-ellipsoidal to ovoid, hyaline, aseptate, smooth-walled, 2.7–5.5 × 1.9–3.4 μm	Solitary or in chains, mostly intercalary, globose to subglobose, hyaline when immature, brown to dark brown when mature, (7.5–)9–25(–32) × (4.8–)7.2–11.2(–13) μm	This study

#### Coniochaeta terricola

Taxon classificationFungiConiochaetalesConiochaetaceae

N. Suwannar., J. Kumla, S. Singsalood & Senwanna sp. nov.

E6F68029-D5EC-5894-86AE-BCA5662ED7E0

863213

Fig. 3

##### Etymology.

“*terricola*” refers to the soil habitat in which this species was isolated.

**Figure 3.**
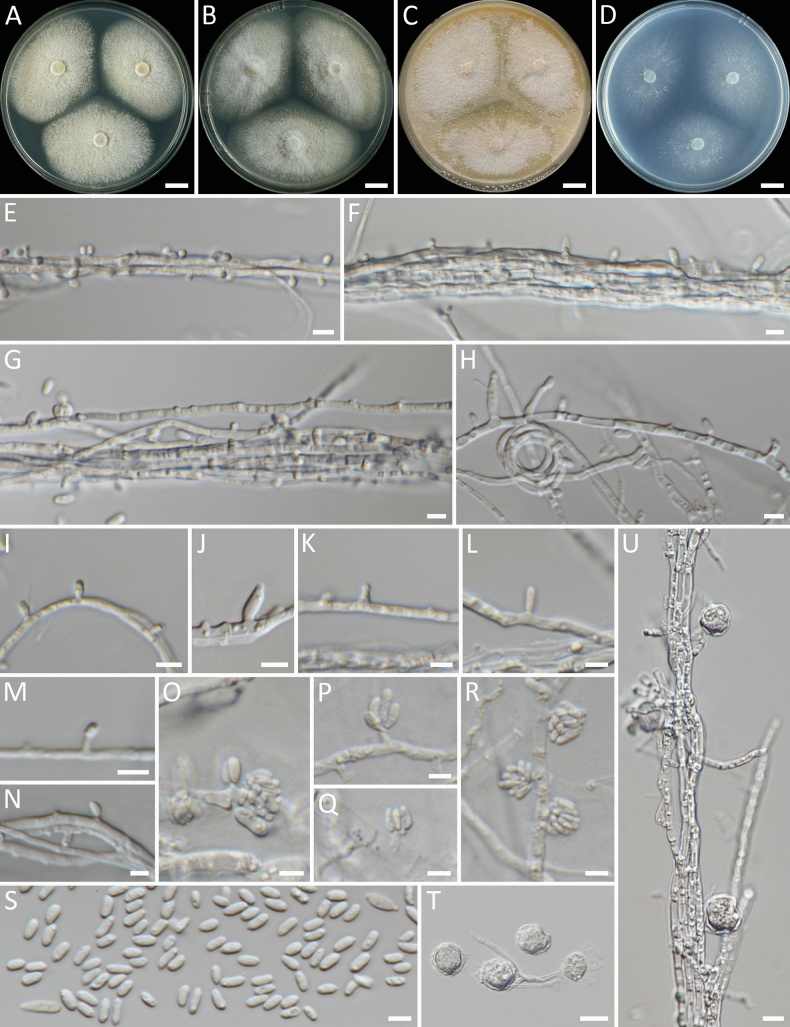
*Coniochaeta terricola* (SDBR-CMU889, ex-type). **A–D**. Colonies on PDA, MEA, OA, and SNA, respectively, after 14 d at 25 °C; **E–G**. Hyphae with conidiogenous cells and conidia; **H**. Hyphal coil and conidiophores; **I–N**. Conidiogenous cells on hyphal cells; **O–R**. Conidiogenous cells and conidia; **S**. Conidia; **T, U**. Chlamydospores. Scale bars: 10 mm (**A–D**), 5 µm (**E–S**), 10 µm (**T, U**).

##### Type.

Thailand • Lampang Province, Mueang Lampang District, Ton Thong Chai Subdistrict, 18°19'09"N, 99°31'42"E, elevation 241 m, isolated from soil, 20 April 2025, N. Suwannarach, S. Singsalood, and J. Kumla, holotype, CMUB40149 (a dried culture grown on PDA), ex-type living culture SDBR-CMU889.

##### Cultural characteristics.

Colonies on different agar media were incubated in the dark at 25 °C for 14 days. On PDA, colonies 39 to 44 mm in diameter, light grey, flat, velvety, with an entire edge; reverse pale yellow. On MEA, colonies 53 to 58 mm in diameter, light grey to light brownish grey, radiating, effuse, and azonate; reverse light grey to pale yellow. On OA, colonies 65 to 70 mm in diameter, light grey to creamy white; reverse creamy white. On SNA, colonies 59 to 65 mm in diameter, creamy white, with immersed mycelia, effuse; reverse white. ***Sexual morph*** Undetermined. ***Asexual morph in vitro*** hyphomycetous. ***Vegetative hyphae*** 1.7–3.3 μm wide, hyaline, septate, branched, thin- and smooth-walled, tightly packed, occasionally forming characteristic hyphal coils and spirally intertwined filaments. ***Conidiophores*** hyaline, short, semi-macronematous, undifferentiated. ***Conidiogenous cells*** phialidic, single or in clusters on short lateral branches, cylindrical, ampulliform or lageniform, separated from subtending hypha by a well-defined basal septum, apical collarette indistinct, hyaline, 2.5–7(–9.7) × 1.4–2.8(–3.6) μm (x̄ = 4.4 × 2.3 μm, *n* = 30). ***Conidia*** ellipsoidal to oblong, rounded at apex and truncate at base, aseptate, hyaline, smooth-walled, 4–6.5(–8.5) × 2–3.6 μm (x̄ = 5.7 × 2.6 μm, *n* = 60; *L/W* ratio = 2.2). ***Chlamydospores*** solitary, terminal or intercalary, subglobose to ovoid, hyaline, 10–15(–18) × 8.5–14 μm (x̄ = 12.9 × 10.8 μm, *n* = 30).

##### Cardinal temperatures for growth on MEA and PDA after two weeks.

Minimum at 10 °C (15 to 24 mm in diameter), 15 °C (30 to 44 mm in diameter), 20 °C (38 to 54 mm in diameter), 25 °C (39 to 58 mm in diameter). Optimum at 30 °C (65 to 75 mm in diameter). Maximum at 37 °C (42 to 58 mm in diameter). No growth was observed at 4 °C and 45 °C.

##### Additional strains examined.

Thailand • Lampang Province, Mueang Lampang District, Ton Thong Chai Subdistrict, 18°19'09"N, 99°31'42"E, elevation 241 m, isolated from soil, 20 April 2025, N. Suwannarach, living cultures SDBR-CMU890 and SDBR-CMU891.

##### Additional GenBank numbers.

SDBR-CMU889; PZ309328 (*tub*), SDBR-CMU890; PZ309329 (*tub*), and SDBR-CMU891; PZ309330 (*tub*).

##### Notes.

BLAST searches of the newly generated sequences from *C. terricola* strains SDBR-CMU889–SDBR-CMU891 against the NCBI nucleotide database revealed that the closest match to the ITS sequences was *C. cymbiformispora* NBRC 32199, with 97.14% similarity. The closest matches to the LSU sequences, with 97.81% similarity, were *C. caraganae* MFLUCC 18-0780 and *C. corticalis* JKI-GP-23-050. The closest match to the *tef1-α* sequences was *C. elegans* FF0093, with 96.54% similarity. In the multilocus phylogenetic tree (Fig. 1), the three strains of *C. terricola* formed a distinct clade sister to *C. angustispora* and *C. cymbiformispora*. A nucleotide comparison of the ITS region between *C. terricola* and *C. angustispora* CBS 144.70 and *C. cymbiformispora* NBRC 32199 revealed 17/576 base-pair differences (2.95%, including gaps) and 15/558 base-pair differences (2.69%, including gaps), respectively, whereas LSU comparisons indicated that *C. terricola* differed from *C. angustispora* CBS 144.70 by 22/896 base-pair differences (2.45%) and from *C. cymbiformispora* NBRC 32199 by 15/583 base-pair differences (2.57%). However, comparison of the *tef1-α* sequences could not be performed because of the lack of available sequence data for *C. angustispora* CBS 144.70 and *C. cymbiformispora* NBRC 32199. Morphologically, *C. terricola* differs from *C. angustispora* in having wider, ellipsoidal to oblong conidia, whereas *C. angustispora* has narrower, subcylindrical to allantoid conidia [4–6.5(–8.5) × 2–3.6 μm vs. 5–8.5 × 1.25–1.75 μm] (Hawksworth and Yip 1981). However, the morphological characteristics of *C. terricola* could not be compared with those of *C. cymbiformispora* because *C. cymbiformispora* is represented only by its sexual morph (Asgari et al. 2007). Therefore, *C. terricola* is established as a novel species based on morphological characteristics and phylogenetic analyses.

## Discussion

Tropical regions, particularly Southeast Asia, are recognized as global biodiversity hotspots; however, fungal taxa in these regions remain insufficiently studied, and their diversity is far from fully documented (Hyde et al. 2018; Corrales et al. 2022; Pham et al. 2025; Pampolina et al. 2026; Tondeleir et al. 2026). Therefore, increased research efforts in tropical regions are essential to uncover hidden fungal diversity, improve taxonomic resolution, and enhance understanding of the ecological roles of fungi. In this study, two new species of *Coniochaeta*, namely *C. saxicola* and *C. terricola*, were discovered in northern Thailand. Their identification was achieved using an integrative approach combining morphological characteristics with multilocus phylogenetic analyses, in accordance with previous studies (Nasr et al. 2018; Harrington et al. 2019; Arnold et al. 2021a, 2021b; Campos-Quiroz et al. 2023). The combined LSU, ITS, and *tef1-α* sequence dataset indicated that *C. saxicola* and *C. terricola* formed distinct clades within the genus *Coniochaeta* (Fig. 1). *Coniochaeta saxicola* clustered as a sister lineage to *C. aurantiaca*, *C. fibrosae*, *C. groatii*, *C. massiliensis*, *C. pulveracea*, *C. teitelbaumiae*, *C. rhopalochaeta*, and *C. geophila*, whereas *C. terricola* grouped as a sister lineage to *C. cymbiformispora* and *C. angustispora*. Comparisons of nucleotide base pairs between *C. saxicola* and its related taxa and between *C. terricola* and its related taxa are presented above (Table 2). Although *tef1-α* sequences have been incorporated into phylogenetic analyses, their relatively low variability and limited taxon coverage provide insufficient resolution for delimiting species boundaries, particularly within the *Coniochaeta* complex (i.e., *C. cephalothecoides*, *C. endophytica*, *C. lutea*, *C. palaoa*, *C. prunicola*, and *C. sordaria*). In addition, the use of different primer sets across studies often amplifies non-overlapping regions of protein-coding genes, reducing comparability among datasets and further limiting species delimitation (Damm et al. 2010; Perdomo et al. 2013; Nasr et al. 2018; Harrington et al. 2019; Arnold et al. 2021a, 2021b; Kabtani et al. 2022). Consequently, beta-tubulin (*tub*) sequence data were excluded from the analyses because they could not be reliably aligned with the dataset used in this study. Although a combination of protein-coding genes is essential for differentiating species complexes and provides strong statistical support, such approaches have been applied in only a few *Coniochaeta* studies (Khan et al. 2013; Nasr et al. 2018; Arnold et al. 2021a, 2021b). The limited availability of protein-coding gene sequences remains a major constraint on species delimitation within the genus.

The morphological characteristics of both *C. saxicola* and *C. terricola* are consistent with the generic concept of *Coniochaeta* described in previous studies (Hawksworth and Yip 1981; Weber 2002; Damm et al. 2010; Khan et al. 2013; Samarakoon et al. 2018; Si et al. 2021). The morphological differences between *C. saxicola* and *C. terricola* and their phylogenetically related taxa are described above. Among the taxa closely related to *C. saxicola* and *C. terricola* (Fig. 1), morphological comparisons could not be made with *C. boothii*, *C. cymbiformispora*, *C. geophila*, *C. pulveracea*, and *C. subcorticalis* because these species were described based on their sexual morph state (Cooke 1887; Munk 1948; Gochenaur 1984; García et al. 2006; Asgari et al. 2007; Forin et al. 2021). Similarly, comparisons with *C. groatii* and *C. teitelbaumiae* were not feasible because of the lack of available morphological data (Tan et al. 2023, 2025a). Therefore, further investigations and sampling of *Coniochaeta* are needed to improve understanding of the genus.

Ecologically, species of *Coniochaeta* are known to occupy a wide range of habitats, including soil, plant debris, and extreme environments characterized by stressful conditions, such as high acidity or wildfire disturbance, suggesting adaptation to diverse environmental conditions (Vázquez-Campos et al. 2014, 2015; Czachura 2024; Philpott et al. 2025). Species of *Coniochaeta* are frequently isolated from soil environments, and some species play functional roles in lignocellulose degradation and the reduction of toxicant levels in plant biomass (Jiménez et al. 2017, 2020; Crous et al. 2025). Additionally, *Coniochaeta* species contribute significantly to nutrient cycling and to the organic matter in ecosystems (Trifonova et al. 2009; Mondo et al. 2019; Nichols et al. 2022; Zhou et al. 2026). Some *Coniochaeta* species have also been reported as occasional opportunistic pathogens in humans and animals (Troy et al. 2013; Kabtani et al. 2022; Pegany et al. 2024; Suchodolski et al. 2025). In Thailand, *C. krabiensis*, a saprobic fungus isolated from submerged marine wood in Phang Nga Province (Dayarathne et al. 2020), and *C. ligniaria*, an endophytic fungus isolated from *Baeckea frutescens* in Loei Province (Kokaew et al. 2011), have been discovered. In this study, *C. terricola* was isolated from soil and *C. saxicola* from rock samples in northern Thailand, highlighting that soil- and rock-associated habitats remain largely unexplored reservoirs of fungal diversity. Future studies should focus on elucidating the functional roles of *Coniochaeta* species in different ecosystems and the effects of global climate change. Moreover, investigating their potential applications in biotechnology and assessing their roles in human and animal health will contribute to a more comprehensive understanding of this diverse genus.

In conclusion, this study integrates morphological characteristics with multilocus phylogenetic analyses to identify two new *Coniochaeta* species from northern Thailand. The findings provide valuable insights for scientists and researchers and are expected to stimulate further investigations of microfungi, particularly *Coniochaeta*, in Thailand. The discovery of these taxa contributes to the understanding of fungal diversity and ecology in tropical ecosystems and emphasizes the need for continued exploration, particularly in understudied regions of Asia. Further studies of these novel fungal species are required to explore their biotechnological potential, particularly with respect to the production of bioactive secondary metabolites, lignocellulolytic enzymes, and other value-added compounds. Furthermore, members of *Coniochaeta* are known to inhabit diverse and sometimes extreme environments; therefore, future studies should focus on genomic analyses of the mechanisms underlying their adaptation to extreme conditions and the impacts of climate change on the diversity and adaptive capacity of this genus.

## Supplementary Material

XML Treatment for Coniochaeta saxicola

XML Treatment for Coniochaeta terricola

## Additional information

### Artificial Intelligence (AI) use

The authors accept full responsibility for the content of the manuscript, including the disclosure of any use of AI.

No AI tools were used in the preparation of this manuscript.

### Author ORCIDs

C. Senwanna https://orcid.org/0000-0002-1008-4514

S. Singsalood https://orcid.org/0009-0005-5726-7042

P. Kodchasee https://orcid.org/0000-0003-0432-4910

S. Tibpromma https://orcid.org/0000-0002-4706-6547

J. Kumla https://orcid.org/0000-0002-3673-6541

N. Suwannarach https://orcid.org/0000-0002-2653-1913

### Data availability

All of the data that support the findings of this study are available in the main text.

## References

[B1] Ahmad S, Johnson RJ, Hillier S, Shelton WR, Rinaldi MG (1985) Fungal peritonitis caused by *Lecythophora mutabilis*. Journal of Clinical Microbiology 22: 182–186. 10.1128/jcm.22.2.182-186.1985 PMC268355 4031032

[B2] Arnold AE, Harrington AH, U’Ren JM, Oita S, Inderbitzin P (2021a) Two new endophytic species enrich the *Coniochaeta endophytica* / *C. prunicola* clade: *Coniochaeta lutea* sp. nov. and *C. palaoa* sp. nov. Plant and Fungal Systematics 66: 66–78. 10.35535/pfsyst-2021-0006

[B3] Arnold AE, Harrington AH, Huang YL, U’Ren JM, Massimo NC, Knight-Connoni V, Inderbitzin P (2021b) *Coniochaeta elegans* sp. nov., *Coniochaeta montana* sp. nov. and *Coniochaeta nivea* sp. nov., three new species of endophytes with distinctive morphology and functional traits. International Journal of Systematic and Evolutionary Microbiology 71: e005003. 10.1099/ijsem.0.005003 34731078

[B4] Asgari B, Zare R, Gams W (2007) *Coniochaeta ershadii*, a new species from Iran, and a key to well-documented *Coniochaeta* species. Nova Hedwigia 84: 175–188. 10.1127/0029-5035/2007/0084-0175

[B5] Bilański P, Kowalski T (2022) Fungal endophytes in *Fraxinus excelsior* petioles and their *in vitro* antagonistic potential against the ash dieback pathogen *Hymenoscyphus fraxineus*. Microbiological Research 257: e126961. 10.1016/j.micres.2022.126961 35042053

[B6] Campos-Quiroz C, Castro JF, Santelices C, Carrasco Fernández J, Guerra M, Cares-Gatica D, Ortiz-Campos J, Ocares Y, Barra-Bucarei L, Theelen B (2023) Description of two fungal endophytes isolated from *Fragaria chiloensis subsp. chiloensis f. patagonica* sp. nov. and a new record of *Coniochaeta hansenii*. Taxonomy 3: 183–203. 10.3390/taxonomy3020014

[B7] Checa J, Barrasa JM, Moreno G, Fort F, Guarro J (1988) The genus *Coniochaeta* (Sacc.) Cooke (*Coniochaetaceae*, *Ascomycotina*) in Spain. Cryptogamie, Mycologie 9: 1–34. 10.5962/p.354247

[B8] Chen KH, Liao HL, Arnold AE, Korotkin HB, Wu SH, Matheny PB, Lutzoni F (2022) Comparative transcriptomics of fungal endophytes in co‐culture with their moss host *Dicranum scoparium* reveals fungal trophic lability and moss unchanged to slightly increased growth rates. New Phytologist 234: 1832–1847. 10.1111/nph.18078 35263447

[B9] Cooke MC (1887) Synopsis pyrenomycetum. Grevillea 16: 16–19.

[B10] Coronado-Ruiz C, Avendaño R, Escudero-Leyva E, Conejo-Barboza G, Chaverri P, Chavarría M (2018) Two new cellulolytic fungal species isolated from a 19^th^-century art collection. Scientific Reports 8: e7492. 10.1038/s41598-018-24934-7 PMC5945893 29748544

[B11] Corrales A, Koch RA, Vasco-Palacios A, Smith M, Ge Z-W, Henkel TW (2022) Diversity and distribution of tropical ectomycorrhizal fungi. Mycologia 114: 919–933. 10.1080/00275514.2022.2115284 36194092

[B12] Crous PW, Carnegie AJ, Wingfield MJ, Sharma R, Mughini G, Noordeloos ME, Santini A, Shouche YS, Bezerra JDP, Dima B, Guarnaccia V, Imrefi I, Jurjević Ž, Knapp DG, Kovács GM, Magistà D, Perrone G, Rämä T, Rebriev YA, Shivas RG, Singh SM, Souza-Motta CM, Thangavel R, Adhapure NN, Alexandrova AV, Alfenas AC, Alfenas RF, Alvarado P, Alves AL, Andrade DA, Andrade JP, Barbosa RN, Barili A, Barnes CW, Baseia IG, Bellanger J-M, Berlanas C, Bessette AE, Bessette AR, Biketova AYu, Bomfim FS, Brandrud TE, Bransgrove K, Brito ACQ, Cano-Lira JF, Cantillo T, Cavalcanti AD, Cheewangkoon R, Chikowski RS, Conforto C, Cordeiro TRL, Craine JD, Cruz R, Damm U, de Oliveira RJV, de Souza JT, de Souza HG, Dearnaley JDW, Dimitrov RA, Dovana F, Erhard A, Esteve-Raventós F, Félix CR, Ferisin G, Fernandes RA, Ferreira RJ, Ferro LO, Figueiredo CN, Frank JL, Freire KTLS, García D, Gené J, Gęsiorska A, Gibertoni TB, Gondra RAG, Gouliamova DE, Gramaje D, Guard F, Gusmão LFP, Haitook S, Hirooka Y, Houbraken J, Hubka V, Inamdar A, Iturriaga T, Iturrieta-González I, Jadan M, Jiang N, Justo A, Kachalkin AV, Kapitonov VI, Karadelev M, Karakehian J, Kasuya T, Kautmanová I, Kruse J, Kušan I, Kuznetsova TA, Landell MF, Larsson K-H, Lee HB, Lima DX, Lira CRS, Machado AR, Madrid H, Magalhães OMC, Majerova H, Malysheva EF, Mapperson RR, Marbach PAS, Martín MP, Martín-Sanz A, Matočec N, McTaggart AR, Mello JF, Melo RFR, Mešić A, Michereff SJ, Miller AN, Minoshima A, Molinero-Ruiz L, Morozova OV, Mosoh D, Nabe M, Naik R, Nara K, Nascimento SS, Neves RP, Olariaga I, Oliveira RL, Oliveira TGL, Ono T, Ordoñez ME, de Ottoni MA, Paiva LM, Pancorbo F, Pant B, Pawłowska J, Peterson SW, Raudabaugh DB, Rodríguez-Andrade E, Rubio E, Rusevska K, Santiago ALCMA, Santos ACS, Santos C, Sazanova NA, Shah S, Sharma J, Silva BDB, Siquier JL, Sonawane MS, Stchigel AM, Svetasheva T, Tamakeaw N, Telleria MT, Tiago PV, Tian CM, Tkalčec Z, Tomashevskaya MA, Truong HH, Vecherskii MV, Visagie CM, Vizzini A, Yilmaz N, Zmitrovich IV, Zvyagina EA, Boekhout T, Kehlet T, Læssøe T, Groenewald JZ (2019) Fungal Planet description sheets: 868–950. Persoonia 42: 291–473. 10.3767/persoonia.2019.42.11 PMC6712538 31551622

[B13] Crous PW, Hernández-Restrepo M, Schumacher RK, Cowan DA, Maggs-Kölling G, Marais E, Wingfield MJ, Yilmaz N, Adan OCG, Akulov A, Duarte EÁ, Berraf-Tebbal A, Bulgakov TS, Carnegie AJ, de Beer ZW, Decock C, Dijksterhuis J, Duong TA, Eichmeier A, Hien LT, Houbraken JAMP, Khanh TN, Liem NV, Lombard L, Lutzoni FM, Miadlikowska JM, Nel WJ, Pascoe IG, Roets F, Roux J, Samson RA, Shen M, Spetik M, Thangavel R, Thanh HM, Thao LD, van Nieuwenhuijzen EJ, Zhang JQ, Zhang Y, Zhao LL, Groenewald JZ (2021) New and Interesting *Fungi*. 4. Fungal Systematics and Evolution 28: 255–343. 10.3114/fuse.2021.07.13 PMC8165967 34124627

[B14] Crous PW, Catcheside DEA, Catcheside PS, Alfenas AC, Alfenas RF, Barreto RW, Lebel T, Balashov S, Broadbridge J, Jurjević Ž, De la Peña-Lastra S, Hoffmann R, Mateos A, Riebesehl J, Shivas RG, Soliz Santander FF, Tan YP, Altés A, Bandini D, Carriconde F, Cazabonne J, Czachura P, Gryta H, Eyssartier G, Larsson E, Pereira OL, Rigueiro-Rodríguez A, Wingfield MJ, Ahmad W, Bibi S, Denman S, Esteve-Raventós F, Hussain S, Illescas T, Luangsa-ard JJ, Möller L, Mombert A, Noisripoom W, Olariaga I, Pancorbo F, Paz A, Piątek M, Polman-Short C, Suárez E, Afshan NS, Ali H, Arzanlou M, Ayer F, Barratt J, Bellanger J-M, Bidaud A, Bishop-Hurley SL, Bohm M, Bose T, Campo E, Chau NB, Çolak ÖF, Cordeiro TRL, Cruz MO, Custódio FA, Couceiro A, Darmostuk V, Dearnaley JDW, de Azevedo Santiago ALCM, Freitas LWS, Yáñez-Morales MJ, Domnauer C, Dentinger B, Dhileepan K, De Souza JT, Dovana F, Eberhardt U, Eisvand P, Erhard A, Fachada V, García-Martín A, Groenewald M, Hammerbacher A, Harms K, Haroon S, Haqnawaz M, Henriques S, Hernández AJ, Jacobus LM, Jaen-Contreras D, Jangsantear P, Kaygusuz O, Knoppersen R, Kumar TKA, Lynch MJ, Mahiques R, Maraia GL, Marbach PAS, Mehrabi-Koushki M, Miller PR, Mongkolsamrit S, Moreau P-A, Oberlies NH, Oliveira JA, Orlovich D, Pérez-Méndez AS, Pinto A, Raja HA, Ramírez GH, Raphael B, Rodrigues A, Rodrigues H, Ramos DO, Safi A, Sarwar S, Saar I, Sánchez RM, Santana JS, Scrace J, Sales LS, Silva LNP, Stryjak-Bogacka M, Tacconi A, Thanh VN, Thomas A, Thuy NT, Toome M, Valdez-Carrazco JM, van Vuuren NI, Vasey J, Vauras J, Vila-Viçosa C, Villarreal M, Visagie CM, Vizzini A, Whiteside EJ, Groenewald JZ (2025) Fungal Planet description sheets: 1781–1866. Persoonia 54: 327–587. 10.3114/persoonia.2025.54.10 PMC12308287 40746709

[B15] Czachura P (2024) *Coniochaeta fodinicola* (*Fungi*: *Sordariomycetes*) from a sulphurous spring in Poland. Plant and Fungal Systematics 69: 7–13. 10.35535/pfsyst-2024-0002

[B16] Damm U, Fourie PH, Crous PW (2010) Coniochaeta (Lecythophora), *Collophora* gen. nov. and *Phaeomoniella* species associated with wood necroses of *Prunus* trees. Persoonia – Molecular Phylogeny and Evolution of Fungi 24: 60–80. 10.3767/003158510X500705 PMC2890157 20664761

[B17] Dayarathne MC, Jones EBG, Maharachchikumbura SSN, Devadatha B, Sarma VV, Khongphinitbunjong K, Hyde KD (2020) Morpho-molecular characterization of microfungi associated with marine based habitats. Mycosphere 11: 1–188. 10.5943/mycosphere/11/1/1

[B18] El-Sayed ES, Grzelczyk J, Strzała T, Gałązka-Czarnecka I, Budryn G, Boratyński F (2025) Bioprospecting endophytic fungi of forest plants for their monoamine oxidase A and cholinesterases inhibitors, and peroxisome proliferator-activated receptor gamma agonists. Journal of Applied Microbiology 136: lxaf034. 10.1093/jambio/lxaf034 39947203

[B19] Forin N, Vizzini A, Fainelli F, Ercole E, Baldan B (2021) Taxonomic re-examination of nine *Rosellinia* types (*Ascomycota*, *Xylariales*) stored in the Saccardo mycological collection. Microorganisms 9: e666. 10.3390/microorganisms9030666 PMC8005106 33807044

[B20] Friebes G, Jaklitsch WM, García S, Voglmayr H (2016) *Lopadostoma taeniosporum* revisited and a new species of *Coniochaeta*. Sydowia 68: 87–97.

[B21] Fu J, Hu L, Shi Z, Sun W, Yue D, Wang Y, Ma X, Ren Z, Zuo Z, Peng G, Zhong Z, Deng J, Cao S, Hu Y (2021) Two metabolites isolated from endophytic fungus *Coniochaeta* sp. F-8 in *Ageratina adenophora* exhibit antioxidative activity and cytotoxicity. Natural Product Research 35: 2840–2848. 10.1080/14786419.2019.1675060 31638432

[B22] García D, Stchigel AM, Cano J, Calduch M, Hawksworth DL, Guarro J (2006) Molecular phylogeny of *Coniochaetales*. Mycological Research 110: 1271–1289. 10.1016/j.mycres.2006.07.007 17081739

[B23] Glass NL, Donaldson GC (1995) Development of primer sets designed for use with the PCR to amplify conserved genes from filamentous ascomycetes. Applied and Environmental Microbiology 61: 1323–1330. 10.1128/aem.61.4.1323-1330.1995 PMC167388 7747954

[B24] Gochenaur SE (1984) *Coniochaeta nepalica*, a common Long Island soil fungus. Mycotaxon 19: 85–92. 10.5962/p.418737

[B25] Hall TA (1999) BioEdit: a user-friendly biological sequence alignment editor and analysis program for Windows 95/98/NT. Nucleic Acids Symposium Series 41: 95–98.

[B26] Han J, Liu C, Li L, Zhou H, Liu L, Bao L, Chen Q, Song F, Zhang L, Li E, Liu L (2017) Decalin-containing tetramic acids and 4-hydroxy-2-pyridones with antimicrobial and cytotoxic activity from the fungus *Coniochaeta cephalothecoides* collected in Tibetan Plateau (Medog). The Journal of Organic Chemistry 82: 11474–11486. 10.1021/acs.joc.7b02010 29019245

[B27] Harrington AH, Del Olmo-Ruiz M, U’Ren JM, Garcia K, Pignatta D, Wespe N, Sandberg DC, Huang YL, Hoffman MT, Arnold AE (2019) *Coniochaeta endophytica* sp. nov., a foliar endophyte from healthy tissue of *Platycladus orientalis*. Plant Fungal Systematics 64: 65–79. 10.2478/pfs-2019-0008

[B28] Hawksworth DL, Yip HY (1981) *Coniochaeta angustispora* sp. nov. from roots in Australia, with a key to the species known in culture. Australian Journal of Botany 29: 377–84. 10.1071/BT9810377

[B29] Hernández-Restrepo M, Gené J, Mena-Portales J, Cano J, Madrid H, Castaneda-Ruiz RF, Guarro J (2014) New species of *Cordana* and epitypification of the genus. Mycologia 106: 723–734. 10.3852/13-122 24891420

[B30] Hou LW, Giraldo A, Groenewald JZ, Rämä T, Summerbell RC, Zang P, Cai L, Crous PW (2023) Redisposition of acremonium-like fungi in *Hypocreales*. Studies in Mycology 105: 23–203. 10.3114/sim.2023.105.02 PMC11182610 38895703

[B31] Huhndorf SM, Miller AN, Fernández FA (2004) Molecular systematics of the *Sordariales*: the order and the family *Lasiosphaeriaceae* redefined. Mycologia 96: 368–387. 10.1080/15572536.2005.11832982 21148859

[B32] Hyde KD, Norphanphoun C, Chen J, Dissanayake AJ, Doilom M, Hongsanan S, Jayawardena RS, Jeewon R, Perera RH, Thongbai B, Wanasinghe DN, Wisitrassameewong K, Tibpromma S, Stadler M (2018) Thailand’s amazing diversity: Up to 96% of fungi in northern Thailand may be novel. Fungal Diversity 93: 215–239. 10.1007/s13225-018-0415-7

[B33] Hyde KD, Dong Y, Phookamsak R, Jeewon R, Bhat DJ, Jones EBG, Liu NG, Abeywickrama PD, Mapook A, Wei DP, Perera RH, Manawasinghe IS, Pem D, Bundhun D, Karunarathna A, Ekanayaka AH, Bao DF, Li JF, Samarakoon MC, Chaiwan N, Lin CG, Phutthacharoen K, Zhang SN, Senanayake IC, Goonasekara ID, Thambugala KM, Phukhamsakda C, Tennakoon DS, Jiang HB, Yang J, Zeng M, Huanraluek N, Liu JK, Wijesinghe SN, Tian Q, Tibpromma S, Brahmanage RS, Boonmee S, Huang SK, Thiyagaraja V, Lu YZ, Jayawardena RS, Dong W, Yang EF, Singh SK, Singh SM, Rana S, Lad SS, Anand G, Devadatha B, Niranjan M, Sarma VV, Liimatainen K, Aguirre‐Hudson B, Niskanen T, Overall A, Alvarenga RLM, Gibertoni TB, Pfliegler WP, Horváth E, Imre A, Alves AL, Santos ACS, Tiago PV, Bulgakov TS, Wanasinghe DN, Bahkali AH, Doilom M, Elgorban AM, Maharachchikumbura SSN, Rajeshkumar KC, Haelewaters D, Mortimer PE, Zhao Q, Lumyong S, Xu JC, Sheng J (2020) Fungal diversity notes 1151–1276: taxonomic and phylogenetic contributions on genera and species of fungal taxa. Fungal Diversity 100: 5–277. 10.1007/s13225-020-00439-5

[B34] Index Fungorum (2026) The Royal Botanic Gardens, Kew. http://www.indexfungorum.org [accessed 27 February 2026]

[B35] Jayawardena RS, Hyde KD, Wang S, Sun Y, Suwannarach N, Sysouphanthong P, Abdel-Wahab MA, Abdel-Aziz FA, Abeywickrama PD, Abreu VP, Armand A, Aptroot A, Bao D, Begerow D, Bellanger J, Bezerra JDP, Bundhun D, Calabon MS, Cao T, Cantillo T, Carvalho JLVR, Chaiwan N, Chen C, Courtecuisse R, Cui B, Damm U, Denchev CM, Denchev TT, Deng CY, Devadatha B, de Silva NI, dos Santos LA, Dubey NK, Dumez S, Ferdinandez HS, Firmino AL, Gafforov Y, Gajanayake AJ, Gomdola D, Gunaseelan S, Shucheng H, Htet ZH, Kaliyaperumal M, Kemler M, Kezo K, Kularathnage ND, Leonardi M, Li J, Liao C, Liu S, Loizides M, Luangharn T, Ma J, Madrid H, Mahadevakumar S, Maharachchikumbura SSN, Manamgoda DS, Martín MP, Mekala N, Moreau P, Mu Y, Pahoua P, Pem D, Pereira OL, Phonrob W, Phukhamsakda C, Raza M, Ren G, Rinaldi AC, Rossi W, Samarakoon BC, Samarakoon MC, Sarma VV, Senanayake IC, Singh A, Souza MF, Souza-Motta CM, Spielmann AA, Su W, Tang X, Tian X, Thambugala KM, Thongklang N, Tennakoon DS, Wannathes N, Wei D, Welti S, Wijesinghe SN, Yang H, Yang Y, Yuan H, Zhang H, Zhang J, Balasuriya A, Bhunjun CS, Bulgakov TS, Cai L, Camporesi E, Chomnunti P, Deepika YS, Doilom M, Duan W, Han S, Huanraluek N, Jones EBG, Lakshmidevi N, Li Y, Lumyong S, Luo Z, Khuna S, Kumla J, Manawasinghe IS, Mapook A, Punyaboon W, Tibpromma S, Lu Y, Yan J, Wang Y (2022) Fungal diversity notes 1512–1610: taxonomic and phylogenetic contributions on genera and species of fungal taxa. Fungal Diversity 117: 1–272. 10.1007/s13225-022-00513-0 PMC9948003 36852303

[B36] Jiménez DJ, Hector RE, Riley R, Lipzen A, Kuo RC, Amirebrahimi M, Barry KW, Grigoriev IV, van Elsas JD, Nichols NN (2017) Draft genome sequence of *Coniochaeta ligniaria* NRRL 30616, a lignocellulolytic fungus for bioabatement of inhibitors in plant biomass hydrolysates. Genome Announcements 5: e01476-16. 10.1128/genomeA.01476-16 PMC5270693 28126934

[B37] Jiménez DJ, Wang Y, Chaib de Mares M, Cortes-Tolalpa L, Mertens JA, Hector RE, Lin J, Johnson J, Lipzen A, Barry K, Mondo SJ, Grigoriev IV, Nichols NN, van Elsas JD (2020) Defining the eco-enzymological role of the fungal strain *Coniochaeta* sp. 2T2.1 in a tripartite lignocellulolytic microbial consortium. FEMS Microbiology Ecology 96: fiz186. 10.1093/femsec/fiz186 31769802

[B38] Jones EG, Devadatha B, Abdel-Wahab MA, Dayarathne MC, Zhang SN, Hyde KD, Liu JK, Bahkali AH, Sarma VV, Tibell S, Tibell L (2020) Phylogeny of new marine *Dothideomycetes* and *Sordariomycetes* from mangroves and deep-sea sediments. Botanica Marina 63: 155–181. 10.1515/bot-2019-0014

[B39] Kabtani J, Militello M, Ranque S (2022) *Coniochaeta massiliensis* sp. nov. isolated from a clinical sampl28. Journal of Fungi 8: e999. 10.3390/jof8100999 PMC9605391 36294564

[B40] Katoh K, Rozewicki J, Yamada KD (2019) MAFFT online service: multiple sequence alignment, interactive sequence choice and visualization. Briefings in Bioinformatics 20: 1160–1166. 10.1093/bib/bbx108 PMC6781576 28968734

[B41] Khan Z, Gené J, Ahmad S, Cano J, Al-Sweih N, Joseph L, Chandy R, Guarro J (2013) *Coniochaeta polymorpha*, a new species from endotracheal aspirate of a preterm neonate, and transfer of *Lecythophora* species to *Coniochaeta*. Antonie Van Leeuwenhoek 104: 243–252. 10.1007/s10482-013-9943-z 23748934

[B42] Kodchasee P, Khunnamwong P, Senwanna C, Duangkon N, Wongsa K, Tiemsan S, Kaewnunta A, Kumla J, Suwannarach N (2026) Anthophilous cryptic yeasts: an integrative polyphasic approach reveals novel taxa in northern Thailand. Mycosphere 17: e002. 10.48130/mycosphere-0026-0002

[B43] Kokaew J, Manoch L, Worapong J, Chamswarng C, Singburaudom N, Visarathanonth N, Piasai O, Strobel G (2011) *Coniochaeta ligniaria* an endophytic fungus from *Baeckea frutescens* and its antagonistic effects against pathogenic fungi. Thai Journal of Agricultural Science 44: 123–131.

[B44] Leonhardt S, Büttner E, Gebauer AM, Hofrichter M, Kellner H (2018) Draft Genome Sequence of the Sordariomycete Lecythophora (Coniochaeta) hoffmannii CBS 245.38. Genome Announcements 6: 10–1128. 10.1128/genomeA.01510-17 PMC5814504 29449401

[B45] Li M, Raza M, Song S, Hou L, Zhang ZF, Gao M, Huang JE, Liu F, Cai L (2023) Application of culturomics in fungal isolation from mangrove sediments. Microbiome 11: e272. 10.1186/s40168-023-01708-6 PMC10712113 38082427

[B46] Liu YJ, Whelen S, Hall BD (1999) Phylogenetic relationships among ascomycetes: evidence from an RNA polymerse II subunit. Molecular Biology and Evolution 16: 1799–1808. 10.1093/oxfordjournals.molbev.a026092 10605121

[B47] Manoharachary C, Rao PR (1973) *Thielavia boothii* sp. nov. from pond mud. Transactions of the British Mycological Society 61: 196–198. 10.1016/S0007-1536(73)80105-6

[B48] Marriott DJ, Wong KH, Aznar E, Harkness JL, Cooper DA, Muir D (1997) *Scytalidium dimidiatum* and *Lecythophora hoffmannii*: unusual causes of fungal infections in a patient with AIDS. Journal of Clinical Microbiology 35: 2949–2952. 10.1128/jcm.35.11.2949-2952.1997 PMC230093 9350765

[B49] Matheny PB, Yajuan JL, Ammirati JF, Hall BD (2002) Using RPB1 sequences to improve phylogenetic inferences among mushrooms (*Inocybe*, *Agaricales*). American Journal of Botany 89: 688–698. 10.3732/ajb.89.4.688 21665669

[B50] Matsushima T (1971) Microfungi of the Solomon Islands and Papua-New Guinea. Published by the Author, Kobe, Japan, 78 pp.

[B51] Miller M, Pfeiffer W, Schwartz T (2010) Creating the CIPRES Science Gateway for inference of large phylogenetic trees. Proceedings of the gateway computing environments workshop (GCE), New Orleans (USA), November, IEEE Publisher, 1–8. 10.1109/GCE.2010.5676129

[B52] Mondo SJ, Jiménez DJ, Hector RE, Lipzen A, Yan M, LaButti K, Barry K, van Elsas JD, Grigoriev IV, Nichols NN (2019) Genome expansion by allopolyploidization in the fungal strain *Coniochaeta* 2T2.1 and its exceptional lignocellulolytic machinery. Biotechnology for Biofuels 12: e229. 10.1186/s13068-019-1569-6 PMC6757388 31572496

[B53] Munk A (1948) *Pyrenomycetes* collected in the peninsula Mols, Jutland. Dansk Botanisk Arkiv 12: 1–19.

[B54] MycoBank (2026) MycoBank. https://www.mycobank.org [Accessed 27.02.2026]

[B55] Nasr S, Bien S, Soudi MR, Alimadadi N, Shahzadeh Fazeli SA, Damm U (2018) Novel *Collophorina* and *Coniochaeta* species from *Euphorbia polycaulis*, an endemic plant in Iran. Mycological Progress 17: 755–771. 10.1007/s11557-018-1382-9

[B56] Nichols NN, Mertens JA, Frazer SE, Hector RE (2022) Growth of *Coniochaeta* species on acetate in biomass sugars. Fermentation 8: e721. 10.3390/fermentation8120721

[B57] Nylander J (2008) MrModeltest2 version 2.3 (programfor selectingDNA substitution models using PAUP*). Evolutionary Biology Centre, Uppsala, Sweden.

[B58] Pampolina NM, Tadiosa ER, Ata JP, Soriano JK, Parlucha JA, Tabao NS, Ha NM, Rahayu S (2026) Biodiversity of Forest *Fungi* and Potential Applications Toward a Sustainable Ecosystem in Southeast Asia Region. In: Guerrero JJG, De Leon MP, Cena-Navarro RB, Notarte KIR, Balendres MAO, dela Cruz TEE (Eds) Mycology in a Changing Planet: Applications and Perspectives from Southeast Asia. Springer, Singapore, 451–485. 10.1007/978-981-95-2640-6_17

[B59] Pegany RB, Wilson RL, Stiefel H, Albert DM, Chamberlain WD, Redd TK (2024) *Coniochaeta hoffmannii* and *mutabilis* invasive fungal keratitis: A case series and literature review. Eye Banking and Corneal Transplantation 3: e0024. 10.1097/ebct.0000000000000024

[B60] Perdomo H, García D, Gené J, Cano J, Sutton DA, Summerbell R, Guarro J (2013) *Phialemoniopsis*, a new genus of *Sordariomycetes*, and new species of *Phialemonium* and *Lecythophora*. Mycologia 105: 398–421. 10.3852/12-137 23099515

[B61] Pham NQ, Marincowitz S, Crous PW, Wingfield MJ (2025) Diversity of soil-borne *Gliocladiopsis* from Indonesia, Malaysia and Vietnam. Fungal Systematics and Evolution 16: 81–92. 10.3114/fuse.2025.16.6 PMC12486216 41041176

[B62] Philpott TJ, Danyagri G, Wallace B, Frank M (2025) Low-severity wildfire prevents catastrophic impacts on fungal communities and soil carbon stability in a fire-affected Douglas-fir ecosystem. Geoderma 454: e117189. 10.1016/j.geoderma.2025.117189

[B63] Preedanon S, Suetrong S, Srihom C, Somrithipol S, Kobmoo N, Saengkaewsuk S, Srikitikulchai P, Klaysuban A, Nuankaew S, Chuaseeharonnachai C, Chainuwong B, Muangsong C, Zhang ZF, Cai L, Boonyuen N (2023) Eight novel cave fungi in Thailand’s Satun Geopark. Fungal Systematics and Evolution 12: 1–30. 10.3114/fuse.2023.12.01 PMC10915585 38455950

[B64] Raja HA, Shearer CA, Fournier J, Miller AN (2012) Freshwater ascomycetes: *Coniochaeta gigantospora* sp. nov. based on morphological and molecular data. Mycoscience 53: 373–380. 10.1007/S10267-012-0181-4

[B65] Rajan YA, Anitha V, Meenakshi R, Kasi M (2023) Therapeutic contact lens-related infection by a novel pathogenic fungus *Coniochaeta hoffmannii*-a case report. Indian Journal of Medical Microbiology 44: e100361. 10.1016/j.ijmmb.2023.02.004 37356829

[B66] Rambaut A (2016) FigTree, version 1.4.3. University of Edinburgh, Edinburgh.

[B67] Réblová M, Miller AN, Rossman AY, Seifert KA, Crous PW, Hawksworth DL, Abdel-Wahab MA, Cannon PF, Daranagama DA, De Beer ZW, Huang S-K, Hyde KD, Jayawardena R, Jaklitsch W, Jones EBG, Ju Y-M, Judith C, Maharachchikumbura SSN, Pang K-L, Petrini LE, Raja HA, Romero AI, Shearer C, Senanayake IC, Voglmayr H, Weir BS, Wijayawar den NN (2016) Recommendations for competing sexual-asexually typified generic names in *Sordariomycetes* (except *Diaporthales*, *Hypocreales*, and *Magnaporthales*). IMA Fungus 7: 131–153. 10.5598/imafungus.2016.07.01.08 PMC4941682 27433444

[B68] Rehner SA, Buckley E (2005) A *Beauveria* phylogeny inferred from nuclear ITS and EF1-α sequences: evidence for cryptic diversification and links to *Cordyceps* teleomorphs. Mycologia 97: 84–98. 10.1080/15572536.2006.11832842 16389960

[B69] Romero AI, Carmarán CC, Lorenzo LE (1999) A new species of *Coniochaeta* with a key to the species known in Argentina. Mycological Research 103: 689–695. 10.1017/S0953756298007564

[B70] Ronquist F, Teslenko M, van der Mark P, Ayres DL, Darling A, Höhna S, Larget B, Liu L, Suchard MA, Huelsenbeck JP (2012) MrBayes 3.2: efficient Bayesian phylogenetic inference and model choice across a large model space. Systematic Biology 61: 539–542. 10.1093/sysbio/sys029 PMC3329765 22357727

[B71] Rosa LH, Queiroz SC, Moraes RM, Wang X, Techen N, Pan Z, Cantrell CL, Wedge DE (2013) *Coniochaeta ligniaria*: antifungal activity of the cryptic endophytic fungus associated with autotrophic tissue cultures of the medicinal plant *Smallanthus sonchifolius* (*Asteraceae*). Symbiosis 60: 133–42. 10.1007/s13199-013-0249-8

[B72] Sakaeyama S, Sano A, Murata Y, Kamei K, Nishimura K, Hatai K (2007) *Lecythophora hoffmannii* isolated from a case of canine Osteomyelitis in Japan. Medical Mycology 45: 72–267. 10.1080/13693780601188602 PMC7537612 17464847

[B73] Samarakoon MC, Gafforov Y, Liu N, Maharachchikumbura SS, Bhat JD, Liu JK, Promputtha I, Hyde KD (2018) Combined multi-gene backbone tree for the genus *Coniochaeta* with two new species from Uzbekistan. Phytotaxa 336: 43–58. 10.11646/phytotaxa.336.1.3

[B74] Sastoque AP, Cano-Lira JF, Stchigel AM (2024) Diversity of rock-inhabiting fungi in Tarragona province, Spain. Journal of Fungi 10: e170. 10.3390/jof10030170 PMC10971471 38535179

[B75] Senanayake IC, Rossi W, Leonardi M, Weir A, McHugh M, Rajeshkumar KC, Verma RK, Karunarathna SC, Tibpromma S, Ashtekar N, Ashtamoorthy SK, Raveendran S, Kour G, Singh A, De la Peña-Lastra S, Mateos A, Kolařík M, Antonín V, Ševčíková H, Esteve-Raventós F, Larsson E, Pancorbo F, Moreno G, Altés A, Turégano Y, Du TY, Lu L, Li QR, Kang JC, Gunaseelan S, Kezo K, Kaliyaperumal M, Fu JZ, Samarakoon MC, Gafforov Y, Teshaboeva S, Kunjan PC, Chamaparambath A, Flakus A, Etayo J, Rodriguez-Flakus P, Zhurbenko MP, de Silva NI, Tennakoon DS, Latha KPD, Manimohan P, Raj KNA, Calabon MS, Ahmadpour A, Heidarian Z, Alavi Z, Alavi F, Ghosta Y, Azizi R, Luo M, Zhao MP, Kularathnage ND, Hua L, Yang YH, Liao CF, Zhao HJ, Lestari AS, Jayasiri SC, Yu FM, Lei L, Liu JW, Karimi O, Tang SM, Sun YR, Wang Y, Zeng M, Htet ZH, Linaldeddu BT, Alves A, Phillips AJL, Bregant C, Montecchio L, De Kesel A, Hustad VP, Miller AN, Fedosova AG, Kučera V, Raza M, Hussain M, Chen YP, Thiyagaraja V, Gomdola D, Rathnayaka AR, Dissanayake AJ, Suwannarach N, Hongsanan S, Maharachchikumbura SSN, Dissanayake LS, Wijayawardene NN, Phookamsak R, Lumyong S, Jones EBG, Yapa N, Wanasinghe DN, Xie N, Doilom M, Manawasinghe IS, Liu JK, Zhao Q, Xu B, Hyde KD, Song JG (2023) Fungal diversity notes 1611–1716: taxonomic and phylogenetic contributions on fungal genera and species emphasis in south China. Fungal Diversity 122: 161–403. 10.1007/s13225-023-00523-6

[B76] Shah S, Shah B, Sharma R, Rekadwad B, Shouche YS, Sharma J, Pant B (2022) Colonization with non-mycorrhizal culturable endophytic fungi enhances orchid growth and indole acetic acid production. BMC Microbiology 22: 101. 10.1186/s12866-022-02507-z PMC9006483 35418028

[B77] Si HL, Su YM, Zheng XX, Ding MY, Bose T, Chang RL (2021) Phylogenetic and morphological analyses of *Coniochaeta* isolates recovered from Inner Mongolia and Yunnan revealed three new endolichenic fungal species. MycoKeys 83: 105–121. 10.3897/mycokeys.83.71140.figure4 PMC8445908 34602840

[B78] Silva RMF, Gonçalves CM, Oliveira TGL, Oliveira RJV, Silva JM, Oliveira MLB, Alves MVS, Silva GA (2023) *Coniochaeta monsterae*, sp. nov. (*Coniochaetaceae*, *Coniochaetales*): a new endophytic fungal species from Brazil. Sydowia 75: 283–292.

[B79] Stamatakis A (2014) RAxML version 8: A tool for phylogenetic analysis and post-analysis of large phylogenies. Bioinformatics 30: 1312–1313. 10.1093/bioinformatics/btu033 PMC3998144 24451623

[B80] Stiller JW, Hall BD (1997) The origin of red algae: Implications for plastid evolution. Proceedings of the National Academy of Sciences of the United States of America 94: 4520–4525. 10.1073/pnas.94.9.4520 PMC20755 9114022

[B81] Suchodolski J, Parol M, Cal-Smok M, Piecuch A, Ogórek R (2025) First report of increased amphotericin B resistance in *Coniochaeta polymorpha* isolates from *Rangifer tarandus platyrhynchus* droppings in Spitsbergen. Fungal Biology 129: e101567. 10.1016/j.funbio.2025.101567 40222767

[B82] Suetrong S, Preedanon S, Kobmoo N, Srihom C, Somrithipol S, Saengkaewsuk S, Srikitikulchai P, Klaysuban A, Nuankaew S, Chuaseeharonnachai C, Chainuwong B, Muangsong C, Malimart K, Rungjindamai N, Siripornpibul C, Chareonkunnatum U, Ploydam B, Thungprue N, Tongsima S, Zhang ZF, Cai L, Boonyuen N (2023) Unravelling the hidden diversity of cave mycobiota in Thailand’s Satun Geopark. Scientific Reports 13: e19162. 10.1038/s41598-023-43316-2 PMC10628224 37932293

[B83] Suwannarach N, Kumla J, Khuna S, Thitla T, Senwanna C, Hongsanan S, Lumyong S (2023) *Anteaglonium saxicola* (*Anteagloniaceae*, *Pleosporales*), a new species isolated from rocks in northern Thailand. Phytotaxa 629: 75–84. 10.11646/phytotaxa.629.1.6

[B84] Suwannarach N, Khuna S, Chaiwong K, Senwanna C, Nuangmek W, Jaturong K (2024) Identification and fungicide sensitivity of the blue mold pathogen in postharvest-stored elephant garlic bulbs in Thailand. Studies in Fungi 9: e015. 10.48130/sif-0024-0015

[B85] Tan YP (2023) Nomenclatural novelties. Index of Australian Fungi 26: 1–6. 10.5281/zenodo.10211858

[B86] Tan YP, Shivas RG (2023) Nomenclatural novelties. Index of Australian Fungi 8: 1–14. 10.5281/zenodo.8042623

[B87] Tan YP, Young AJ, Campbell SD, Peart JR (2023) Nomenclatural novelties. Index of Australian Fungi 20: 1–5. 10.5281/zenodo.10035658

[B88] Tan YP, Bishop-Hurley SL, Conroy JR, Shivas RG (2025a) Nomenclatural novelties. Index of Australian Fungi 50: 1–12. 10.5281/zenodo.14693768

[B89] Tan YP, Dearnaley JDW, Mapperson RR (2025b) Nomenclatural novelties. Index of Australian Fungi 51: 1–11. 10.5281/zenodo.14829467

[B90] Taniguchi Y, Taketani T, Moriyama H, Moriki S, Nishimura K, Sato E, Notsu Y, Higuchi T, Sugitani Y, Yasuda K, Nagai A, Yamaguchi S, Shibata H, Masuda J (2009) Septic shock induced by *Lecythophora mutabilis* in a patient with mitochondrial encephalomyopathy. Journal of medical microbiology 58: 1255–1258. 10.1099/jmm.0.010678-0 19528157

[B91] Tondeleir L, Amalfi M, Stubbe D, Verbeken A (2026) Tales of the unexpected II: two new angiocarpic representatives of *Russulaceae* (*Russulales*, *Basidiomycota*) from tropical Southeast Asia. MycoKeys 129: 213–239. 10.3897/mycokeys.129.177637 PMC12988430 41836325

[B92] Trifonova R, Postma J, Van Elsas JD (2009) Interactions of plant‐beneficial bacteria with the ascomycete *Coniochaeta ligniaria*, Journal of Applied Microbiology 106: 1859–1866. 10.1111/j.1365-2672.2009.04163.x 19298515

[B93] Troy GC, Panciera DL, Pickett JP, Sutton DA, Gene J, Cano JF, Guarro J, Thompson EH, Wickes BL (2013) Mixed infection caused by *Lecythophora canina* sp. nov. and *Plectosphaerella cucumerina* in a German shepherd dog. Medical Mycology 51: 455–460. 10.3109/13693786.2012.754998 23294425

[B94] van Heerden A, van Zyl WH, Cruywagen CW, Mouton M, Botha A (2011) The lignicolous fungus *Coniochaeta pulveracea* and its interactions with syntrophic yeasts from the woody phylloplane. Microbial Ecology 62: 609–619. 10.1007/s00248-011-9869-9 21590300

[B95] Vázquez-Campos X, Kinsela AS, Waite TD, Collins RN, Neilan BA (2014) *Fodinomyces uranophilus* gen. nov. sp. nov. and *Coniochaeta fodinicola* sp. nov., two uranium mine-inhabiting *Ascomycota* fungi from northern Australia. Mycologia 106: 1073–1089. 10.3852/14-013 25143478

[B96] Vázquez-Campos X, Kinsela AS, Collins RN, Neilan BA, Aoyagi N, Waite TD (2015) Uranium binding mechanisms of the acid-tolerant fungus *Coniochaeta fodinicola*. Environmental Science & Technology 49: 8487–96. 10.1021/acs.est.5b01342 26106944

[B97] Vilgalys R, Hester M (1990) Rapid genetic identification and mapping of enzymatically amplified ribosomal DNA from several *Cryptococcus* species. Journal of Bacteriology 172: 4238–4246. 10.1128/jb.172.8.4238-4246.1990 PMC213247 2376561

[B98] Vu D, Groenewald M, De Vries M, Gehrmann T, Stielow B, Eberhardt U, Al-Hatmi A, Groenewald JZ, Cardinali G, Houbraken J, Boekhout T, Crous PW, Robert V, Verkley GJM (2019) Large-scale generation and analysis of filamentous fungal DNA barcodes boosts coverage for kingdom fungi and reveals thresholds for fungal species and higher taxon delimitation. Studies in Mycology 92: 135–54. 10.1016/j.simyco.2018.05.001 PMC6020082 29955203

[B99] Wanasinghe DN, Phukhamsakda C, Hyde KD, Jeewon R, Lee HB, Gareth Jones EB, Tibpromma S, Tennakoon DS, Dissanayake AJ, Jayasiri SC, Gafforov Y, Camporesi E, Bulgakov TS, Ekanayake AH, Perera RM, Samarakoon MC, Goonasekara ID, Mapook A, Li WJ, Senanayake IC, Li J, Norphanphoun C, Doilom M, Bahkali AH, Xu J, Mortimer PE, Tibell L, Tibell S, Karunarathna SC (2018) Fungal diversity notes 709–839: Taxonomic and phylogenetic contributions to fungal taxa with an emphasis on fungi on *Rosaceae*. Fungal Diversity 89: 1–236. 10.1007/s13225-018-0395-7

[B100] Wang Q, Wang C, Wei Y, Yao W, Lei Y, Sun Y (2022) Soil microbes drive the flourishing growth of plants from *Leucocalocybe mongolica* fairy ring. Frontiers in Microbiology 13: e893370. 10.3389/fmicb.2022.893370 PMC9164162 35668763

[B101] Weber E, Görke C, Begerow D (2002) The *Lecythophora*-*Coniochaeta* complex II. Molecular studies based on sequences of the large subunit. Nova Hedwig 74: 187–200. 10.1127/0029-5035/2002/0074-0187

[B102] Weber EG (2002) The *Lecythophora*-*Coniochaeta* complex: I. Morphological studies on *Lecythophora* species isolated from *Picea abies*. Nova Hedwig 74: 85–159. 10.1127/0029-5035/2002/0074-0159

[B103] White TJ, Bruns T, Lee S, Taylor J (1990) Amplification and direct sequencing of fungal ribosomal RNA genes for phylogenetics. In: Innis MA, Gelfand DH, Sninsky JJ, White TJ (Eds) PCR Protocols: a Guide to Methods and Amplifications. Academic Press, San Diego, 315–322. 10.1016/B978-0-12-372180-8.50042-1

[B104] Xie J, Strobel GA, Feng T, Ren H, Mends MT, Zhou Z, Geary B (2015) An endophytic *Coniochaeta velutina* producing broad spectrum antimycotics. Journal of Microbiology 53: 390–397. 10.1007/s12275-015-5105-5 26025171

[B105] Zare R, Asgari B, Gams W (2010) The species of *Coniolariella*. Mycologia 102: 1383–1388. 10.3852/10-035 20943542

[B106] Zhang G-Q, Suwannarach N, Disayathanoowat T, Shoocongdej R, Domethong V, Dai D-Q, Liu R-M, Wijayawardene NN, Kumla J (2026) Two new soil-inhabiting fungi, *Malbranchea cavernicola* (*Onygenales*, *Malbrancheaceae*) and *Talaromyces pangmaphaensis* (*Eurotiales*, *Trichocomaceae*), from a cave in northern Thailand. MycoKeys 131: 211–234. 10.3897/mycokeys.131.188256 PMC13129548 42079509

[B107] Zhang T, Wei XL, Wei YZ, Liu HY, Yu LY (2016) Diversity and distribution of cultured endolichenic fungi in the Ny-Ålesund Region, Svalbard (High Arctic). Extremophiles 20: 461–470. 10.1007/s00792-016-0836-8 27240672

[B108] Zheng W, Yang C, Bu W, Wang H, Ma T, Bu F (2026) Age-dependent variations in leaf-endophytic microbiota and biocontrol potential in *Camellia oleifera*. Scientific Reports 16: 1430. 10.1038/s41598-025-31248-y PMC12796465 41350800

[B109] Zhou Y, Wu P, Qu Y, Pang S, Mu H, Yang M, Lin X, Wang D (2026) Layered nitrogen application drives changes in nitrogen nutrition status of wheat by affecting key soil microbial clusters. European Journal of Agronomy 175: 128011. 10.1016/j.eja.2026.128011

